# Multimodal Characterization of Seizures in Zebrafish Larvae

**DOI:** 10.3390/biomedicines10050951

**Published:** 2022-04-20

**Authors:** Lapo Turrini, Michele Sorelli, Giuseppe de Vito, Caterina Credi, Natascia Tiso, Francesco Vanzi, Francesco Saverio Pavone

**Affiliations:** 1 Department of Physics and Astronomy, University of Florence, Via G. Sansone 1, 50019 Sesto Fiorentino, Italy; sorelli@lens.unifi.it; 2 European Laboratory for Non-Linear Spectroscopy, Via Nello Carrara 1, 50019 Sesto Fiorentino, Italy; devito@lens.unifi.it (G.d.V.); credi@lens.unifi.it (C.C.); fvanzi@lens.unifi.it (F.V.); 3 Department of Neuroscience, Psychology, Drug Research and Child Health, University of Florence, Viale Pieraccini 6, 50139 Florence, Italy; 4 National Institute of Optics, National Research Council, Via Nello Carrara 1, 50019 Sesto Fiorentino, Italy; 5 Department of Biology, University of Padova, Via U. Bassi 58/B, 35131 Padova, Italy; natascia.tiso@unipd.it; 6 Department of Biology, University of Florence, Via Madonna del Piano 6, 50019 Sesto Fiorentino, Italy

**Keywords:** epilepsy, seizure, zebrafish, calcium imaging, two-photon, light-sheet microscopy, behavior, tracking, functional connectivity

## Abstract

Epilepsy accounts for a significant proportion of the world’s disease burden. Indeed, many research efforts are produced both to investigate the basic mechanism ruling its genesis and to find more effective therapies. In this framework, the use of zebrafish larvae, owing to their peculiar features, offers a great opportunity. Here, we employ transgenic zebrafish larvae expressing GCaMP6s in all neurons to characterize functional alterations occurring during seizures induced by pentylenetetrazole. Using a custom two-photon light-sheet microscope, we perform fast volumetric functional imaging of the entire larval brain, investigating how different brain regions contribute to seizure onset and propagation. Moreover, employing a custom behavioral tracking system, we outline the progressive alteration of larval swim kinematics, resulting from different grades of seizures. Collectively, our results show that the epileptic larval brain undergoes transitions between diverse neuronal activity regimes. Moreover, we observe that different brain regions are progressively recruited into the generation of seizures of diverse severity. We demonstrate that midbrain regions exhibit highest susceptibility to the convulsant effects and that, during periods preceding abrupt hypersynchronous paroxysmal activity, they show a consistent increase in functional connectivity. These aspects, coupled with the hub-like role that these regions exert, represent important cues in their identification as epileptogenic hubs.

## 1. Introduction

Epilepsy is one of the most common and widespread neurological disorders, affecting nearly 50 million people worldwide [1]. The hallmarks of this pathology are represented by recurrent and unprovoked episodes of paroxysmal neuronal activity, namely seizures, typically occurring as a consequence of a loss of balance between excitatory and inhibitory synaptic communication inside the brain. The etiology underlying this unbalance can be manifold. In primary epilepsies, the loss of balance typically stems from genetic mutations affecting neuronal membrane ion channels or altering neuronal migration or protein degradation in neurons [2,3,4]. In secondary or acquired epilepsies, instead, the unbalance has its origin in a variety of central nervous system (CNS) insults such as brain tumors, traumatic brain injury or stroke [5,6,7,8,9]. Furthermore, acquired epilepsy commonly develops as a comorbidity in patients with neurodegenerative conditions (such as Alzheimer’s, Parkinson’s, and Huntington’s disease [10,11,12], to cite just a few) and some molecular pathways partially overlap [13,14,15]. To this framework we must add that, despite the decrease in the disease burden from 1990 to present [1], epilepsy is still an important cause of disability and mortality. Indeed, even with a large panel of anti-epileptic drugs (AEDs) currently available [16], approximately 30% of patients still suffer from drug-resistant seizures [17].

Besides clinical studies in human patients, the use of animal models brought terrific contributions in understanding the mechanisms ruling epileptic aberrant activity. Particularly, rodent models, coupled with functional imaging techniques, gave important insights for the characterization of the spatial features of epileptic seizures propagation [18,19,20]. However, because of the presence of the bone skull (like most vertebrates) and due to their brain size, rodents allow optical access, at least with current technology, only to limited cortical or sub-cortical regions, thus failing to provide a benchmark for reconstructing the whole picture.

In the past two decades, a model organism, zebrafish (*Danio rerio*), has been making its way into the field of epilepsy research [21]. The larval form of this freshwater teleost thanks to its small size, tissue transparency, high grade of homology with the human genome and ease of genetic handling represents now the vertebrate of choice for neuroscience studies aiming at revealing brain-wide dynamics. Moreover, owing to its external fertilization and rapid development, zebrafish larvae are suitable for high-throughput behavioral tests aiming at screening large chemical libraries for promising candidates as AEDs [22,23,24].

Thus far, several genes and their mutations involved in the pathogenesis of human epilepsy have been studied in zebrafish models [24,25,26,27,28]. Besides genetic models, however, pharmacological ones remain the most popular. Indeed, since the seminal work by Baraban and colleagues [29], several studies have confirmed that pentylenetetrazole (PTZ), a GABA_A_ receptor antagonist, can reliably induce acute seizures in zebrafish larvae, making this pharmacological approach suitable for studying different kinds of human epilepsies in this animal model [30,31,32,33]. Neuroscientists using zebrafish can now rely on an ever-expanding genetic toolbox [34,35] for perturbing and monitoring neuronal activity [36,37], as well as on ad hoc optical methods for whole-brain recording [38] and interactive brain atlases [39,40]. Among optical methods, light-sheet microscopy [41] deserves a place of honor. Indeed, owing to its native optical sectioning and parallelization of the acquisition process within each frame, this technique allows for rapid high-resolution volumetric functional imaging of the transparent larval brain [42,43].

Here, employing a custom two-photon light-sheet microscope and a transgenic zebrafish line expressing the calcium indicator GCaMP6s in all neurons, we performed fast whole-brain functional imaging during PTZ-induced seizures. We characterized the functional modification occurring in the larval brain as a consequence of the convulsant effects and described the kinetic features of calcium transients in four diverse neuronal activity regimes. Moreover, we performed high-speed tracking of freely-swimming larvae to produce a behavioral characterization of the motor outcome associated with different grades of epileptic seizures.

## 2. Materials and Methods

### 2.1. Zebrafish Larvae

For whole-brain imaging experiments we employed n = 12, 4 dpf *Tg(elavl3:H2B-GCaMP6s)* [44] zebrafish larvae in homozygous albino background [45], expressing the fluorescent calcium indicator GcaMP6s in the nuclei of differentiated neurons. Whole-brain calcium imaging data were recorded for our previous work [43]. For behavioral measurements, we used n = 16, 4 dpf *wt* AB/Tübingen larvae. Zebrafish strains were reared according to standard procedures [46].

### 2.2. Two-Photon Light-Sheet Microscope

Whole-brain functional imaging was performed using a custom two-photon light-sheet microscope, as described previously [43]. Briefly, a tunable Ti-Sa pulsed laser (Chameleon Ultra II, Coherent, Santa Clara, CA, USA) operated at 930 nm is used as an excitation source. After a pre-compensation step (PreComp, Coherent), the infrared (IR) beam is adjusted in power and conveyed through an electro-optical modulator (EOM, 84502050006, Qioptiq, Rhyl, UK) which inverts light polarization between two orthogonal states at a frequency of 100 kHz. Light is then routed to a pair of galvanometric mirrors. One mirror in the pair is a resonant one (CRS-8 kHz, Cambridge Technology, Bedford, MA, USA) and it is used to produce a virtual light-sheet, scanning the beam along the rostro-caudal direction of the larva. The other is a closed-loop one (6215H, Cambridge Technology) and it is employed to displace the sheet of light along the dorso-ventral direction of the larva. A polarizing beam splitter diverts the incoming light alternatively, according to its polarization state, to either of the two excitation objectives (XLFLUOR4X/340/0,28, Olympus, Tokyo, Japan). In order to maximize fluorescence excitation, right after the beam splitter a half-wave plate is used to rotate the light polarization plane so that the light from both the excitation objectives is polarized parallel to the table surface [47]. IR light-sheet is focused inside a fish water-filled custom sample chamber, thermostated at 28.5 °C. The fine positioning of the sample chamber under the detection objective (XLUMPLFLN20XW, Olympus, NA = 1) is performed using three micro-positioning stages (M-521.DD1, M-511.DD1 and M-501.1DG, Physik Instrumente, Karlsruhe, Germany). The 20X high-NA detection objective collects the fluorescence signal. After a demagnification step bringing the final magnification of the image to 3×, the fluorescence signal opportunely filtered (FF01-510/84-25 nm BrightLine^®^ single-band bandpass filter, Semrock, Rochester, NY, USA) is routed onto the chip of a sCMOS camera (ORCA-Flash4.0 V3, Hamamatsu Photonics, Hamamatsu, Japan). Before the camera, an electrically tunable lens (ETL; EL-16-40-TC-VIS-5D-C, Optotune, Dietikon, Switzerland) is employed to perform remote focusing of the detection focal plane, in sync with the closed-loop galvo displacing the light-sheet. The system and the acquisition process are controlled via custom software written in “G” (LabVIEW, National Instruments, Austin, TX, USA).

### 2.3. Behavioral Tracking System

We developed a behavioral system specifically designed for high-speed tracking of zebrafish larvae (Figure S1). We designed a custom round behavioral arena, having 3d-printed walls (a transparent stereolithographic resin ring, inner diameter: 45 mm; height: 3 mm) glued into a 9 cm transparent Petri dish (Sarstedt, Nümbrecht, Germany) and a circular cover glass (diameter: 50 mm; thickness: 0.17 mm) on top. A custom annular illumination employing IR LEDs (850 nm, SFH4655, Osram, Munich, Germany) is used to illuminate the arena through its transparent walls. Below the arena, an HD LED display (NHD-7.0-HDMI-HR-RSXP, NewHaven Display International, Elgin, IL, USA) is used to present the larvae with static or dynamic images. Above the arena, a CMOS camera (Blackfly S3, BFS-U3-16S2M-CS, FLIR, Wilsonville, OR, USA), equipped with a wide-angle lens (2.8–10 mm, A4Z2812CS-MPIR, Computar, Cary, NC, USA) and an IR band-pass filter (835/70, FF01-835/70-25, Semrock), is used to record the movement of the larva inside the arena at 300 Hz. Images are streamed, via USB3 connection, to a workstation (Dell, Round Rock, TX, USA) and processed in real-time using a custom software based on the Stytra open-source package [48], to perform live tracking of larval movements. The behavioral setup has a temperature control system, to maintain environmental conditions stable during the measurement.

### 2.4. Whole-Brain Functional Imaging

To perform whole-brain imaging, larvae were mounted in agarose gel as previously described [43]. Briefly, each larva was paralyzed with a solution of d-tubocurarine (2 mM, 10 min; 93750, Sigma-Aldrich, St. Louis, MO, USA) to avoid movement artefacts, included in 1.5% (*w*/*v*) low gelling temperature agarose (A9414, Sigma-Aldrich) in fish water (150 mg/L Instant Ocean, 6.9 mg/L NaH_2_PO_4_, 12.5 mg/L Na_2_HPO_4_, pH 7.2), then mounted on a custom-made glass support and immersed in fish water thermostated at 28.5 °C.

Each larva was imaged for 5 min in control conditions (fish water) before adding the convulsant agent PTZ (P6500, Sigma-Aldrich) at the desired final concentration (1.0, 2.5, 7.5, and 15 mM). Stock solutions of PTZ were prepared by dissolving it in deionized water, while the final concentrations used in the experiments were obtained by diluting the stock in fish water. After PTZ exposure, we imaged seizure onset and propagation alternating 5 min of acquisition every 10 min for 6 cycles (30 min of seizure monitoring over 1 h). According to our previous studies on this pathological model [22,43], we deemed 1 h of brain activity monitoring sufficient to map the development of seizure dynamics. Employing a laser power of approximately 70 mW on the sample, we performed whole-brain volumetric imaging (31 brain planes along 150 μm depth) at 5 Hz, with a voxel size of 2.2 × 2.2 × 5.0 μm^3^.

### 2.5. Behavioral Recordings

Each larva was placed in the behavioral arena, which was filled with fish water (approximately 5 mL) and closed with a coverglass. Behavioral recordings started after 10 min of habituation (at 28.5 °C). The recording was performed in the dark, with IR illumination on to allow tracking, and the display under the arena presenting a white (RGB: 200, 200, 200) uniform background. After 5 min of baseline tracking, fish water inside the arena was replaced with PTZ solution at one of the four final concentrations (1.0, 2.5, 7.5, and 15 mM, in fish water). 30 and 60 min after exposure to the convulsant, each larva underwent two additional 5-min behavioral recordings. In between the recordings, the arenas containing larvae were kept in an incubator at 28.5 °C under uniform illumination.

### 2.6. Data Analysis

#### 2.6.1. Whole-Brain Calcium Imaging

Preprocessing of whole-brain calcium imaging data was performed as we previously described [43]. Briefly, after manually removing frames affected by movement artifacts, for each recording we computed the *ΔF/F*_0_ signal using a custom pixel-based routine. *F_0_* was calculated as the pixel-based first decile value along the temporal dimension.

Then, using ImageJ, we manually drew the boundaries of each anatomical region, thus identifying in each larval brain 10 volumetric regions of interest (ROIs). We then computed the average over time of pixel gray values within each ROI. Employing a custom Matlab (MathWorks Inc., Natick, MA, USA) script, each *ΔF/F*_0_ trace was then detrended (function *msbackadj*, window size: 200 samples; step size: 100 samples) and smoothed using a Savitzky-Golay filter (function *sgolay*, polynomial order: 7; window length: 31 samples).

For calcium peak characterization, after an automatic peak (local maxima) detection step performed in Matlab (function *findpeaks*, min peak amplitude: 2%; min peak prominence 1%; min peak distance: 1 s), we manually processed detrended *ΔF/F*_0_ traces to locate the starting and ending points of each activity peak. Using a custom Matlab script, we calculated the following parameters for each annotated peak: prominence (difference between the highest and lowest values), duration, rise time and decay time. Distributions of peaks belonging to different brain activity regimes were pooled across brain regions and normalized on the total peak count of each activity phase (ctrl, preictal, ictal and postictal). Non-detrended and non-smoothed *ΔF/F*_0_ traces coming from different brain regions were divided into the four different activity regimes and used to compute pairwise correlation coefficients to produce correlation matrices.

#### 2.6.2. Behavioral Recordings

Analysis of kinematic parameters of swimming dynamics was performed using OriginPro 2021 (OriginLab Corp., Northampton, MA, USA). Coordinates of larval head position over time were recovered from Stytra behavioral logs and processed as follows. Larval interframe displacement Δ*d* was computed applying the Pythagorean theorem to consecutive pairs of pixel coordinates ($\Delta d=\sqrt{{\left(x_{i}-x_{i-1}\right)}^{2}+{\left(y_{i}-y_{i-1}\right)}^{2}}$), and then converted into mm (pixel size: 0.06 mm). The distance traveled during each 5-min tracking experiment was computed summing all the Δ*d* of the same recording exceeding a threshold (1.5 μm) to eliminate of localization jitter during larval rest periods. The percentage of time spent in movement was calculated by summing all the time intervals associated with movement and dividing it by the overall duration of the recording. To calculate the swimming speed module, we applied the Pythagorean theorem using as inputs the *x* and *y* components (v*_x_* and v*_y_*, respectively) of the velocity vector v. Subsequently, we performed an automatic velocity peak detection (window: 0.066 s; threshold: 1%), and then computed the average maximum speed in each recording. To calculate the average maximum acceleration, we repeated the same automatic peak detection procedure on the first derivative of the velocity time series. We then counted the number of bouts per recording (automatic peak detection in the larval displacement over time, window: 0.066s; threshold: 2%) and computed the bout frequency (n° of bouts/measurement duration), the bout duration (overall time spent in movement/n° of bouts) and the bout displacement (total distance traveled/n° of bouts).

### 2.7. Statistical Analysis

OriginPro 2021 (OriginLab Corp.) was used to carry out all statistical analyses. Unless otherwise stated, results were considered statistically significant if their corresponding *p*-value was less than 0.05.

We performed both intra-group and inter-group statistical evaluation of behavioral data and frequency of neuronal activity peaks (*ΔF/F*_0_). In detail, intra-group analysis was conducted by applying one-way repeated measures, ANOVA (factor: time of exposure to PTZ; subject: larvae), while inter-group analysis was based on a two-way repeated measures ANOVA (factor A: PTZ concentration; factor B: time of exposure to PTZ; subject: larvae). In both analyses, post-hoc comparisons were performed by employing Tukey’s method.

Statistical comparisons of distributions of peak duration, prominence, and rise/decay times in the four different regimes were performed with the two-sample Kolmogorov-Smirnov test (K-S test), applying Bonferroni’s correction (α = 0.05/6 = 0.00833). K-S test with Bonferroni’s correction (α = 0.05/45 = 0.00111) was also employed for statistical comparisons of the distributions of the same features among the ten different brain regions during ictal regime.

## 3. Results

### 3.1. Zebrafish Brain Regions Present Different Susceptibility to Convulsant Effects

**Figure 1:**
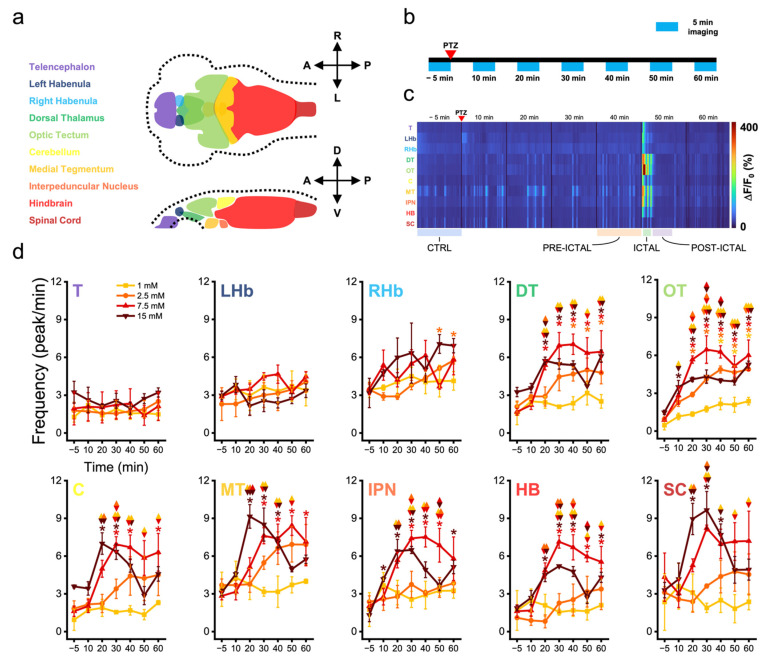
Zebrafish brain regions present different susceptibility to convulsant effects. (**a**) Dorsal (**upper**) and lateral (**lower**) schemes representing in different colors the ten brain districts into which volumetric calcium imaging data were segmented for further analysis. Dotted lines show the outer larval boundaries. A: anterior; P: posterior; R: right; L: left; D: dorsal; V: ventral. (**b**) Scheme representing the brain functional imaging protocol adopted. After PTZ treatment, larvae were imaged for 5 min every 10, along 60 min. (**c**) Map showing in color-code the neuronal activity (*ΔF/F*_0_) of one larva over time of each of the ten brain regions (colored as in panel (**a**)) along 60 min of seizure mapping. Warmer colors indicate higher activity. Below the map, the different brain activity regimes, discussed in Figure 2, are reported. (**d**) Plot showing for each of the ten brain regions (abbreviations and colors as in panel (**c**)) the frequency of activity peaks at different PTZ concentrations (1.0, 2.5, 7.5 and 15 mM, see legend for colors) over 60 min of activity monitoring. Values represent mean ± s.e.m. of n = 3 larvae per concentration. * indicates *p*-value < 0.05 for intra-group comparison with respect to control (−5 min) values, one-way repeated measures ANOVA and post hoc Tukey’s test (see Table S1 for *p*-values). Diamond symbol indicates *p*-value < 0.05 for inter-group comparison at the same time point, two-way repeated measures ANOVA and post hoc Tukey’s test (Table S2). Color couples in each symbol denote compared groups.

To investigate the neuronal dynamics underlying seizure onset and propagation in zebrafish larvae, we performed fast whole-brain functional imaging on a transgenic line expressing the calcium reporter GCaMP6s in all neuronal nuclei. Functional imaging was performed employing a custom light sheet microscope using non-linear excitation. The use of IR light to produce the light sheet, being invisible to this species [49] (as well as to most vertebrates), allowed for measuring neuronal activity without visually perturbing the organism during the acquisition. We recorded fluorescence calcium dynamics at 5 Hz volumetric rate, thus mapping at high-speed the activity arising from ten zebrafish brain districts (Figure 1a). Each larva was first imaged in physiological conditions (i.e., before PTZ treatment) to acquire a baseline recording and was then exposed to PTZ at one of the following concentrations: 1.0, 2.5, 7.5 and 15 mM (Figure 1b). The effects of the convulsant on larval brain activity were monitored for one hour, performing 5 min of whole-brain recording every 10 min (Figure 1b). Figure 1c shows the color-coded neuronal activity over time coming from the different brain regions of a larva exposed to 15 mM PTZ. At 50 min upon the exposure to the convulsant we can see the emergence of high amplitude (warmer colors) hypersynchronous neuronal activity, typically termed ictal activity [50].

We first characterized the effect of the different convulsant concentrations on neuronal activity. Particularly, we investigated how the convulsant affected the frequency of neuronal activation (Figure 1d, Tables S1 and S2). We found the most rostral brain regions (telencephalon, left and right habenula) to be not significantly affected by the convulsant, at any of the concentrations. The lowest concentration tested (1 mM) had no marked effect on all brain regions except for the optic tectum, where we observed a significantly increasing shift after 40 min of exposure with respect to control conditions (Figure 1d, OT, yellow). The same effect was observed in larvae exposed to 2.5 mM PTZ. In this case, the convulsant produced a significant increase in the frequency of calcium activity in both optic tectum and dorsal thalamus, after 20 and 40 min of exposure, respectively (Figure 1d, OT and DT, orange). At the higher concentrations the scenario dramatically changed. Indeed, exposing animals to 7.5 mM PTZ produced a higher and more precocious increase in calcium peak frequency involving not only the optic tectum and the dorsal thalamus, as previously described for the lower concentrations, but also all other brain regions (except for the three more rostral districts). The increase in frequency induced by this concentration appeared to be significant as early as after a 20-min exposure in the optic tectum and dorsal thalamus (Figure 1d, OT and DT, red), and a 30-min exposure in the cerebellum, medial tegmentum, interpeduncular nucleus, and hindbrain (Figure 1d, C, MT, IPN and HB, red). The frequency increase then rapidly reached a plateau and remained stable for the rest of the observation (60 min). Differently from the submaximal convulsant concentration, 15 mM PTZ did not produce a plateauing of activity frequency, but a rapid achievement of the maximal effect and a subsequent decay at longer exposure times. Indeed, maximal PTZ concentration induced a significant increase in the frequency of activity peaks with respect to control, as early as after 10 min of exposure in the optic tectum and interpeduncular nucleus (Figure 1d, OT and IPN, brown), and after 20-min exposure in all other caudal regions. Except for the optic tectum, remaining regions showed a significant tendency to decrease their activation frequency at longer exposures (40 min: C; 50 min: C, DT, MT, IPN, HB and SC; 60 min: C, MT, and SC, brown).

### 3.2. Zebrafish Brain Undergoes Transition between Different Activity Regimes during Seizures

**Figure 2:**
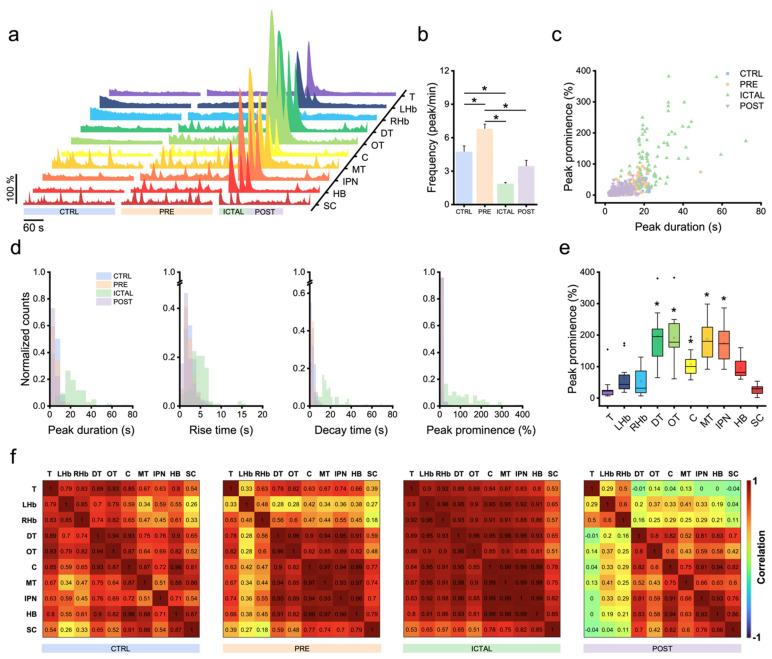
Zebrafish brain undergoes transition between different activity regimes during seizures. (**a**) *ΔF/F*_0_ traces of the ten larval brain regions (colored as in Figure 1a) during four diverse activity regimes observed at maximal PTZ concentration (15 mM). CTRL (pre-exposure), PRE (preictal), ICTAL and POST (postictal). (**b**) Average frequency of activity peaks in the different regimes presented in panel (**a**). * indicates *p*-value < 0.05 for inter-regime comparison, one-way repeated measures ANOVA and post hoc Tukey’s test (for *p*-values see Table S3). Values represent mean ± s.e.m of n = 3 larvae per concentration. (**c**) Scatter plot showing peak duration as a function of peak prominence for each of the four regimes. (**d**) Normalized distributions of calcium transients’ kinetic features (peak duration, rise time, decay time and prominence) in each of the four activity regimes. For each parameter, all four distributions appear significantly different (two-samples K-S test with Bonferroni correction, α = 0.00833), except for peak prominence of CTRL vs. PRE and POST. For *p*-values, see Table S4. (**e**) Peak prominence during ictal regime for each of the ten brain regions. * indicates *p*-value < 0.00111 for inter-region comparison with telencephalon, two-samples K-S test with Bonferroni correction, α = 0.00111 (for *p*-values see Table S5). Box: IQR; error bar: 1.5 IQR; black line: median; white square: mean; black diamond: outlier. (**f**) Correlation matrices reporting average pairwise Pearson’s correlation coefficients of neuronal activity during the four regimes across the ten brain regions.

Since only in larvae exposed to the maximal concentration of the convulsant we observed the presence of ictal events (Figure 1c), we moved the focus of our analysis to the characterization of the distinct brain activity regimes detectable in animals exposed to 15 mM. Figure 2a shows typical *ΔF/F*_0_ traces coming from the ten brain regions during four functional regimes. In detail, we isolated a baseline regime in pre-exposure conditions (Figure 2a, CTRL), a regime preceding ictal activity (Figure 2a, PRE), the emergence of high-amplitude ictal events (Figure 2a, ICTAL) followed by a postictal regime (Figure 2a, POST). With respect to baseline regime, preictal activity was characterized by a significant increase in frequency (Figure 2b, Table S3; CTRL: 4.7 ± 0.5 peak/min; PRE: 6.8 ± 0.4 peak/min). With the emergence of ictal activity, we instead found a significant decrease in frequency compared both to control and preictal regimes (ICTAL: 1.9 ± 0.1 peak/min). During postictal activity, frequency increased again, despite not significantly compared both to control and ictal regimes (POST: 3.5 ± 0.5 peak/min).

Figure 2c shows a scatter plot reporting peak duration as a function of peak prominence for activity peaks isolated in each of the four functional regimes described before. Calcium peaks during ictal activity stood out greatly from the populations of other regimes, owing to their high amplitudes and long durations. On the other hand, while preictal peaks presented values of prominence and duration intermediated between control and ictal ones, postictal peaks appeared to be the population more squeezed towards lower values of both parameters.

Figure 2d shows the normalized distributions of critical features characterizing calcium transients. For each of the parameters analyzed (peak duration, rise/decay times, and peak prominence), we found the population of ictal peaks to have the distributions significantly more shifted towards higher values (Table S4). Indeed, the distribution of ictal peak durations had a median value (21.2 s, IQR 9.6 s) considerably larger from those of other regimes (CTRL: 5.4 s, IQR 3.4 s; PRE: 4.4 s, IQR 2.8 s, POST: 3.4 s, IQR 2.6 s). The same was observed for the rise time (ICTAL: 4.5 s, IQR 2.2 s; CTRL: 2.2 s, IQR 1.6 s; PRE: 2.0 s, IQR 1.4 s, POST: 1.6 s, IQR 1.4 s) and decay time (ICTAL: 15.1 s, IQR 8.3 s; CTRL: 3.0 s, IQR 2.8 s; PRE: 2.2 s, IQR 1.6 s, POST: 1.8 s, IQR 1.9 s). Also, the distribution of ictal peaks prominence had significantly higher values (median 83.6%, IQR 131.6%) with respect to other regimes (CTRL: 3.6%, IQR 3.9%; PRE: 3.8%, IQR 3.5%, POST: 3.3%, IQR 3.7%). Moreover, this last distribution appeared much more stretched with respect to those of other features of the same functional regime (Figure 2d). This was due to the great variability of activity peak prominence arising from different brain regions (Figure 2e). Indeed, with respect to the other features (peak duration, rise and decay times), which did not present significant differences between regions (Figure S2), peak prominence showed significantly higher values in the dorsal thalamus, optic tectum, cerebellum, medial tegmentum, and interpeduncular nucleus (Table S5). Moreover, the activity peaks belonging to control, pre-, and postictal regimes had distribution of kinetic features significantly different from each other (Figure 2d and Figure S3 and Table S4), confirming the existence of distinct activity regimes.

Finally, we investigated how the functional connectivity between brain regions is altered by the dimming effect that the convulsant exerts on inhibitory tone. While submaximal PTZ concentrations (1.0, 2.5, and 7.5 mM) induced a highly heterogeneous panel of functional alterations across animals (Figure S4), thus revealing the high inter-individual susceptibility to the convulsant, the same was not during the different activity regimes of larvae exposed to maximal PTZ concentration. Figure 2f shows average correlation matrices of the four regimes. With respect to physiological conditions (CTRL), in the preictal regime we observed a considerable increase in correlation between the activity of midbrain (dorsal thalamus, optic tectum, cerebellum, medial tegmentum and interpeduncular nucleus) and hindbrain regions. With the appearance of ictal activity, functional connectivity increased dramatically in all regions, as expected during hypersynchronous paroxysmal events. In the postictal regime instead, we observed the polarization of correlation into two groups showing high intra-group and low inter-group functional connectivity: from one side the rostralmost regions (telencephalon and left/right habenula), and from the other the caudalmost ones.

### 3.3. Zebrafish Larvae Undergoes Alteration of Their Swimming Kinematic during Seizures

**Figure 3:**
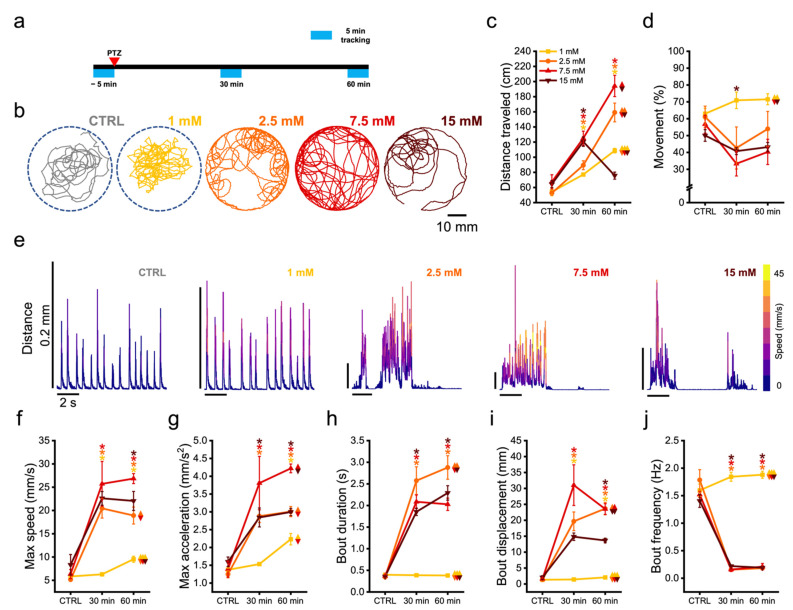
Zebrafish larvae undergo alteration of their swimming kinematic during seizures. (**a**) Scheme representing the behavioral tracking protocol adopted. The larvae were tracked along 5 min before the exposure to PTZ and 30–60 min after. (**b**) Swimming trajectories over 5 min of behavioral recording of one larva for each of the following conditions: pre-exposure (CTRL) and 60 min after the exposure to the different PTZ concentrations. (**c**,**d**) Average total distance traveled (**c**) and percentage of time spent in movement (**d**) in 5 min recording in the 3 different tracking windows (CTRL, 30 and 60 min), in larvae exposed to one of the four PTZ concentrations tested (see legend for colors). (**e**) Representative swimming bout shapes observed in each of the conditions reported, presented as larval displacement over time. Traces are color-mapped according to swimming velocity (warmer colors higher speed). (**f**–**j**) Average kinematic features of swimming bouts -maximum speed (**f**), maximum acceleration (**g**), duration (**h**), displacement (**i**) and frequency (**j**)—during 5-min recording epochs before PTZ exposure and 30–60 min after. * indicates *p*-value < 0.05 for intra-group comparison with respect to control (CTRL) values, one-way repeated measures ANOVA and post hoc Tukey’s test (for *p*-values see Table S6). Diamond symbol indicates *p*-value < 0.05 for inter-group comparison at 60 min exposure, two-way repeated measures ANOVA and post hoc Tukey’s test (for *p*-values see Table S7). Color couples in each symbol indicate groups compared. Values represent mean ± s.e.m. of n = 4 larvae per concentration.

To characterize the changes in behavior associated with different grades of seizures, we performed tracking of freely swimming larvae exposed to the four PTZ concentrations tested in whole-brain imaging experiments. Using a custom system specifically developed to perform behavioral recordings on zebrafish larvae while maintaining stable environmental conditions, we tracked at 300 Hz the movements of each animal along 5 min of swimming inside the behavioral arena, first in physiological conditions, and then after 30 and 60 min upon the exposure to the convulsant (Figure 3a). Figure 3b shows representative trajectories over 5 min of recording, during spontaneous exploration of the arena (in gray) and 1 h upon PTZ treatments (colored traces). We first quantified the total distance traveled by each animal (Figure 3c). While, during control recording, larvae swam on average 59.7 ± 3.1 cm (average value of all 16 larvae), any of the concentrations of PTZ brought to a significant increase in the overall distance traveled 30 min upon administration (1 mM: 77.1 ± 1.8 cm; 2.5 mM: 89.4 ± 5.9 cm; 7.5 mM: 125.1 ± 9.3 cm; 15 mM: 120.1 ± 6.2 cm). 60 min upon PTZ treatment, larvae exposed to any of the concentrations except for the highest, showed a further significant increase in distance swum with respect to control (1 mM: 108.7 ± 3.0 cm; 2.5 mM: 159.2 ± 12.4 cm; 7.5 mM: 194.2 ± 14.1 cm, Table S6). Indeed, 1 h after exposure to 15 mM PTZ, during the period of recording larvae covered a distance not considerably different from that of internal controls (15 mM: 75.7 ± 4.7 cm). Moreover, comparing the distance traveled at 60 min, we found it to be statistically significant among the four different treatments, with larvae exposed to the submaximal PTZ concentration (7.5 mM) showing highest values (Table S7). On average, larvae spent 57.7 ± 2.5% (average value of all 16 animals) of the recording time during control measurement (Figure 3d). Notably, while 30 and 60 min after the exposure to 1 mM PTZ we observed a slight (not significant) increase in this parameter, larvae treated with all other concentrations presented instead a decrease in the percentage of time spent in movement (statistically significant with respect to control only at 30 min for 15 mM, Table S6). After 60 min of exposure to 1 mM PTZ, larvae spent significantly more time in movement with respect to those exposed either to 7.5 or 15 mM (Table S7).

We thus outlined the swimming performances from a finer point of view by quantifying the swim bout kinematic features. Figure 3e highlights in ten-second extracts the typical bout shapes (distance traveled over time, color-coded according to swimming velocity) observed in the different tested conditions. Particularly, during control we observed a regular sequence of closely paced, stereotyped dart-like bouts, typical of exploratory behavior (Video S1). In the presence of 1 mM PTZ, larvae exhibited the same type of bouts as control, despite presenting increased peak velocity (Video S2). On the contrary, at the higher concentrations, the scenario progressively and inexorably changed. Indeed, already at 2.5 mM, larvae tended to lose their physiological swimming posture (dorsal portion facing upwards) and showed rapid eye movements (Video S3). Single-bout behavior was replaced by periods of continuous swimming, characterized by even more enhanced swimming velocity at 7.5 mM (Video S4). Conversely, at 15 mM PTZ larvae exhibited intense uncoordinated movements interspersed by long intervals of no swim (Video S5).

In terms of velocity, the exposure to the convulsant brought an overall increase in maximum bout speed (Figure 3f) with respect to that reached during control (6.4 ± 0.6 mm/s, average value of all 16 larvae). At 30 min, larvae exposed to any of the concentrations, except the lowest, presented a significant increase in maximum velocity with respect to control (1 mM: 6.3 ± 0.2 mm/s; 2.5 mM: 20.5 ± 2.1 mm/s; 7.5 mM: 25.7 ± 4.8 mm/s; 15 mM: 22.6 ± 1.5 mm/s), which remained high after 60 min (1 mM: 9.5 ± 0.6 mm/s; 2.5 mM: 18.9 ± 1.8 mm/s; 7.5 mM: 26.9 ± 1.1 mm/s; 15 mM: 22.1 ± 2.0 mm/s, Table S6). Comparing the maximum bout speed of groups treated with different concentrations after 60-min exposure, we found it to be statistically significant at all the concentrations with respect to the lowest one, with larvae treated with 7.5 mM PTZ showing the highest peak velocity (Table S7). The increasing trend of maximum bout speed reflected that of maximum bout acceleration (Figure 3g). Indeed, average bout acceleration of 1.4 ± 0.1 mm/s^2^ registered during control raised significantly for all concentrations (except 1 mM) at 30-min exposure (1 mM: 1.5 ± 0.1 mm/s^2^; 2.5 mM: 2.9 ± 0.2 mm/s^2^; 7.5 mM: 3.8 ± 0.7 mm/s^2^; 15 mM: 2.8 ± 0.3 mm/s^2^). After 60 min larvae exposed to any condition showed a significant increase in acceleration with respect to control (1 mM: 2.2 ± 0.2 mm/s^2^; 2.5 mM: 3.0 ± 0.1 mm/s^2^; 7.5 mM: 4.2 ± 0.1 mm/s^2^; 15 mM: 3.0 ± 0.1 mm/s^2^, Table S6). When comparing the maximum bout acceleration between treatments at 60-min time point, we found it significantly larger for 7.5 mM with respect to all other concentrations (Table S7). Bout duration (Figure 3h), which was highly conserved during control (0.37 ± 0.01 s, average over all 16 larvae) due to the stereotyped larval exploratory behavior, increased dramatically after 30 min of exposure to the higher concentrations of convulsant (2.5 mM: 2.6 ± 0.3 s; 7.5 mM: 2.1 ± 0.2 s; 15 mM: 1.8 ± 0.1s), with larvae treated with 2.5 mM PTZ showing the highest bout durations. After 1 h of exposure the duration of bouts remained high, with larvae exposed to 2.5 mM showing significantly longer bouts with respect to all other conditions (Table S6). The lowest concentration (1 mM), instead, did not significantly affect the duration of bouts, even after 60 min of exposure (1mM, 30 min: 0.39 ± 0.03 s; 60 min: 0.38 ± 0.02 s). The displacement produced within each bout (Figure 3i), on average 1.58 ± 0.2 mm (value averaged over all 16 larvae) in physiological conditions, was strongly increased by the higher concentrations after 30 min exposure (2.5 mM: 19.7 ± 2.9 mm; 7.5 mM: 31.0 ± 6.4 mm; 15 mM: 14.8 ± 1.2 mm), while it was not affected by the lowest (1 mM: 1.4 ± 0.1 mm). After 1 h of treatment, we observed a slight reduction in the bout average displacement in larvae treated with 7.5 mM and a statistically significant increase in those treated with 1 mM PTZ (Table S6).

Finally, we computed the frequency of swimming bouts. In control conditions larvae produced on average 1.58 ± 0.06 bout/s, while in larvae exposed to 1 mM PTZ this frequency slightly increased over time (30 min: 1.84 ± 0.08 Hz; 60 min: 1.88 ± 0.06 Hz). Conversely, all other concentrations produced a significant drop in swimming events (30 min: 2.5 mM, 0.15 ± 0.03 Hz; 7.5 mM, 0.16 ± 0.03 Hz; 15 mM, 0.22 ± 0.01 Hz. 60 min: 2.5 mM, 0.18 ± 0.02 Hz; 7.5 mM, 0.21 ± 0.06 Hz; 15 mM, 0.19 ± 0.02 Hz) with respect to control (Table S6).

## 4. Discussion

We adopted a multimodal approach, comprising both whole-brain functional imaging and behavioral tracking, to characterize seizures of different grades in zebrafish larvae. Particularly, we employed a custom two-photon light sheet microscope [43,51,52] to record at high speed (5 Hz) the neuronal activity of zebrafish larvae pan-neuronally expressing the calcium indicator GCaMP6s. The system, employing non-visible light as an excitation source, guarantees avoidance of visual perturbation of the animal during the acquisition process, thus returning reliable neuronal activity measurements in a highly sensitive system such as an epileptic brain. Furthermore, the use of IR illumination, owing to its tissue penetration abilities compared to visible light, allows for considerable reduction of undesired striping artifacts in light-sheet microscopy [53]. With this custom system we performed a thorough mapping of the larval encephalon which allowed us to investigate how brain regions are differently recruited during the onset and propagation of PTZ-induced seizures. Besides, we employed a custom behavioral system to track rapid larval movements, and thus producing a description of the kinematic features of larval swimming during seizures.

We wondered to which extent the activity arising from diverse cerebral districts could be impaired by the effect of the convulsive drug. Given the mechanism of action of PTZ, which blocks inhibitory GABAergic synapses [54], we expected the larval brain to increase its firing rate upon drug administration. We thus characterized the resulting calcium activity in terms of frequency of fluorescence peaks. We found that brain regions present diverse susceptibility to convulsant effects. Interestingly, at the lowest PTZ concentration (1 mM) only the optic tectum shows a significant increase in activity. At 2.5 mM, in addition to optic tectum, dorsal thalamus too shows a significant activity enhancement. At the higher concentrations (7.5 and 15 mM), all regions except for the rostralmost (telencephalon and left/right habenula) show a significant rise in neuronal activity. Taken together, these results suggest that at increasing convulsant concentrations ever more brain regions are recruited into seizure generation, starting from midbrain regions and spanning to more caudal ones, with the optic tectum and telencephalon representing the most and the least sensitive district, respectively. These observations are complementary to what was recently presented by Diaz Verdugo et al. [55], who found the optic tectum and the telencephalon to show respectively the larger and the smaller changes in activity during the preictal period.

From a behavioral point of view, the effect of PTZ at its lowest concentration (1 mM) induces an increase in maximum speed and acceleration (and consequently in distance traveled, bout frequency being equal to control), while maintaining a bout shape identical to that of physiological exploratory behavior. Higher convulsant concentrations (2.5 and 7.5 mM) bring instead to dramatic increase in bout velocity, displacement, and duration with a radical modification of swimming behavior, changing from dart-like single-bouts routine to longer periods of uninterrupted uncoordinated movement. Notably, only larvae exposed to 2.5 mM PTZ show a peculiar behavior characterized by rapid eye movements.

Furthermore, increasing PTZ concentrations produce a coherently ever earlier significant enhancement of neuronal spiking in all brain regions, with activity frequency peaking at ever lower exposure times. Interestingly, at maximal concentration, where the frequency peak is reached as early as after 10-min exposure, we observe basically in all regions a drop in spiking frequency at longer exposure times (50–60 min). This phenomenon can be explained by the emergence, only in animals treated with 15 mM PTZ, of alternating neuronal activity regimes. Indeed, baseline activity in physiological conditions (ctrl) is replaced by a higher frequency activity preceding ictal events (preictal). Abruptly the scenario changes with the emergence of high amplitude and long-lasting calcium transients (as we previously observed [22,43]) typical of ictal regime, which are then followed by periods of reduced activity (postictal). Indeed, precisely the presence of alternating ictal and postictal activity accounts for the drop in spiking frequency we observe at longer exposure times, as well as for the dramatic reduction in distance traveled found in behavioral recordings. Indeed, at maximal PTZ concentration, we observe a decrease in distance traveled and in percentage of time spent in movement which are likely to be attributed to both the uncoordinated (and thus not effective) swimming during ictal events and to motionless postictal periods. This result is in contrast with what described by Baraban and colleagues [29], who found 15 mM PTZ to increase the amount of time larvae spent in movement. The discrepancy may arise from the fact that in Baraban’s work larvae during baseline recording (no PTZ) remain basically still.

As we recently described [43], ictal activity spans the entire brain from most caudal to more rostral regions in what we termed a caudo-rostral ictal wave (CRIW). The propagation of this wave-like pattern involves in a different manner diverse brain regions, with midbrain ones (namely, dorsal thalamus, optic tectum, medial tegmentum, and interpeduncular nucleus) showing the highest increase in the prominence of activity peaks. This aspect could be explained by the hub-like function exerted by larval midbrain [56,57,58]. Indeed, those hub regions have manifold incoming connections which could be responsible for tens to hundreds of action potential trains giving rise to high prominence peaks in calcium activity [59].

Furthermore, from a functional connectivity point of view, before the massive increase in correlation observed during ictal discharge (in line with what our group and others previously described [22,43,60]), which completely overrules physiological connectivity mechanisms, midbrain and hindbrain regions show an increased level of correlation already during preictal regime. This confirms and expands what recently described by Diaz Verdugo and colleagues [55]. Indeed, the increase in synchronization observed in preictal activity could identify in midbrain regions putative epileptogenic hubs, which could act as a drive for the abrupt emergence of global ictal discharges. Interestingly, during postictal activity, we observed a sort of polarization of functional connectivity into two separate groups of regions (the rostralmost ones—telencephalon and left/right habenula—and the caudalmost ones—midbrain, hindbrain, and spinal cord), showing low connectivity between each other and thus confirming the identification of a separate postictal regime.

Future studies on epileptic convulsions are needed to shed further light on the role of midbrain districts on seizure onset and propagation. Moreover, employing novel techniques—such as targeted optogenetic stimulation [61]—could aim at interrupting ictal discharges [62], thus reverting the larval brain to different activity regimes.

## Data Availability

Data are available upon reasonable request to the corresponding authors.

## Supplementary Materials

The following supporting information can be downloaded at: https://www.mdpi.com/article/10.3390/biomedicines10050951/s1. Figure S1. Scheme of the behavioral tracking system. Figure S2. Normalized distributions of peak duration, rise and decay time and peak prominence, for each of the four indicated brain activity regimes, shown in Figure 2d. For p-values calculated with two-sample K-S test (Bonferroni correction a = 0.00833) see Table S4. Figure S3. Box charts showing peak duration, rise and decay time for each of the ten brain regions (colored as in Figure 1a) during ictal regime. Peak duration and decay time are not significantly different between regions. Significant difference in rise time is limited to optic tectum (OT) with respect to spinal cord (SC). For p-values calculated with two-sample K-S test (Bonferroni correction a = 0.00111), see Table S5. Figure S4. Correlation matrices reporting Pearson’s correlation coefficients of neuronal activity over the time of exposure to submaximal PTZ concentrations (1.0, 2.5, and 7.5 mM) across brain regions as in Figure 2 (telencephalon, left habenula, right habenula, dorsal thalamus, optic tectum, cerebellum, medial tegmentum, interpeduncular nucleus, hindbrain, and spinal cord). Table S1. Intra-group (same PTZ concentration) comparison of peak frequency at the different measured time points (Figure 1c), compared to pre-exposure (−5 min) values. One-way repeated measures ANOVA followed by Tukey’s test. Colored cells indicate *p*-values < 0.05. Table S2. Inter-group (different PTZ concentrations) comparison of peak frequency at the same measured time points (Figure 1c). Two-way repeated measures ANOVA followed by Tukey’s test. Colored cells indicate *p*-values < 0.05. Table S3. Inter-regime comparison of peak frequency in the different brain activity regimes at 15 mM exposure (Figure 2b). One-way repeated measures ANOVA followed by Tukey’s test. Colored cells indicate *p*-values < 0.05. Table S4. Inter-regime comparison of activity peak features (duration, rise/decay time and prominence) distributions (Figure 2d and Figure S2). Two-samples K-S test with Bonferroni correction. Colored cells indicate *p*-values < 0.00833. Table S5. Inter-region comparison of activity peak features (duration and rise/decay time) during ictal regime (Figure 2e and Figure S3). Two-samples K-S test with Bonferroni correction. Colored cells indicate *p*-values < 0.00111. Table S6. Intra-group (same PTZ concentration) comparison of swimming kinematic features (distance traveled, percentage of time spent in movement, bout max velocity, bout max acceleration, bout, bout duration, bout displacement and bout frequency) presented in Figure 3c,d,f–j, compared to pre-exposure (CTRL) values. One-way repeated measures ANOVA followed by Tukey’s test. Colored cells indicate *p*-values < 0.05. Table S7. Inter-group (different PTZ concentrations) comparison of swimming kinematic features (distance traveled, percentage of time spent in movement, bout max velocity, bout max acceleration, bout, bout duration, bout displacement and bout frequency) presented in Figure 3c,d,f–j, at 60-min PTZ exposure. Two-way repeated measures ANOVA followed by Tukey’s test. Colored cells indicate *p*-values < 0.05. Video S1. Exploratory behavior in physiological conditions (CTRL). Video S2. Swimming behavior after 60-min exposure to 1 mM PTZ. Video S3. Eye movement behavior after 30-min exposure to 2.5 mM PTZ. Video S4. Swimming behavior after 60-min exposure to 7.5 mM PTZ. Video S5. Swimming behavior after 60-min exposure to 15 mM PTZ.
