# Supplementary material for: Multimodal Characterization of Seizures in Zebrafish Larvae
Source: Biomedicines. 2022 Apr 20;10(5):951. doi: 10.3390/biomedicines10050951 (PMC9139036; doi:10.3390/biomedicines10050951)
Supplement: Supplementary file 1 [file biomedicines-10-00951-s001.zip › biomedicines-1649483-supplementary.pdf]

Supplementary Materials

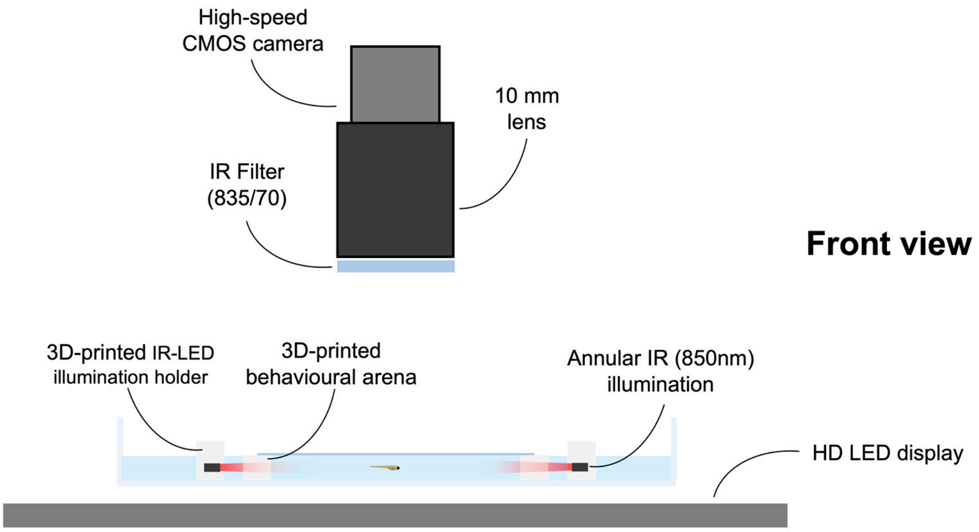

Figure S1. Scheme of the behavioral tracking system.

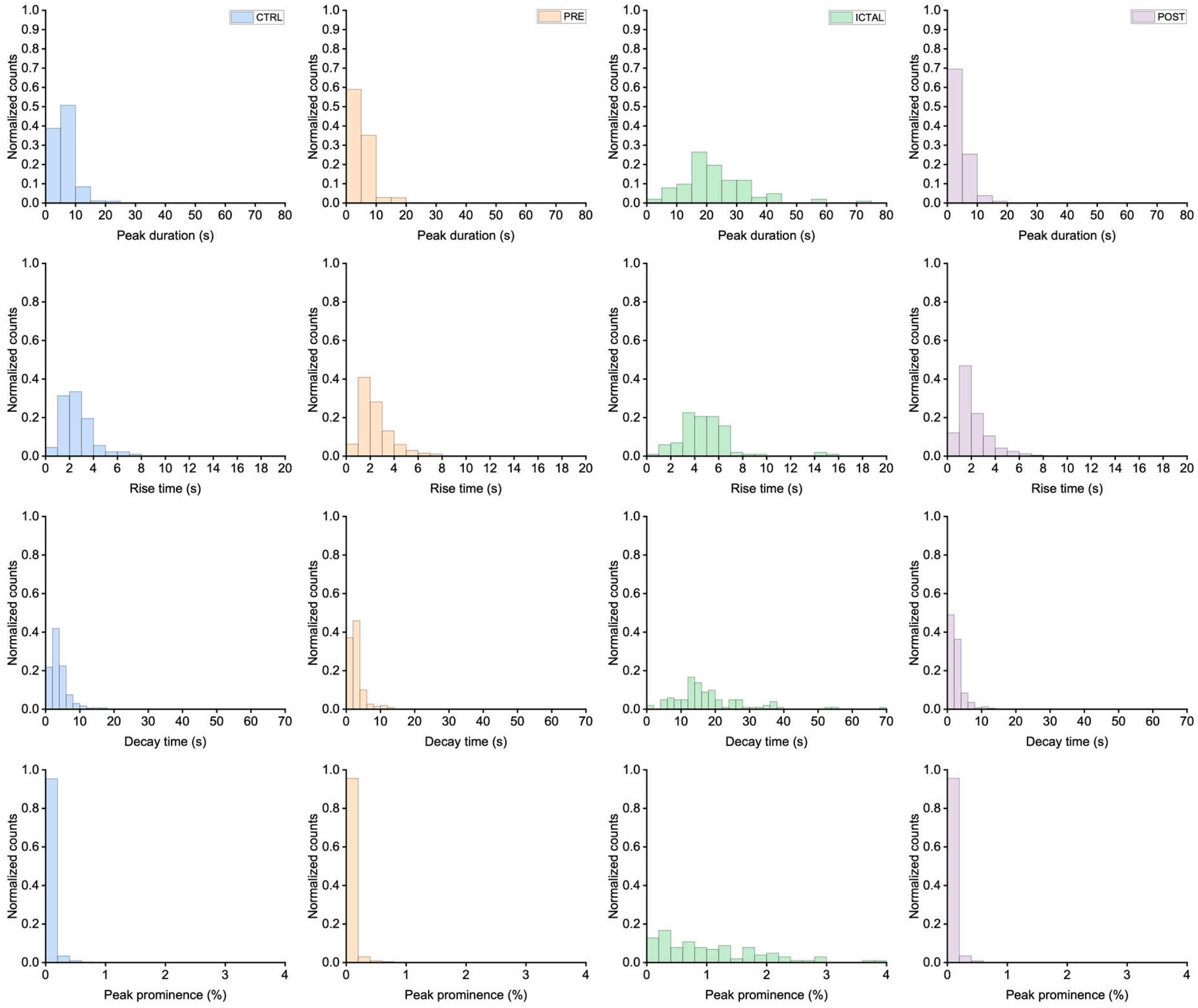

Figure S2. Normalized distributions of peak duration, rise and decay time and peak prominence, for each of the four indicated brain activity regimes, shown in Figure 2d. For p-values calculated with two-sample K-S test (Bonferroni correction $\alpha = 0.00833$) see Table S4.

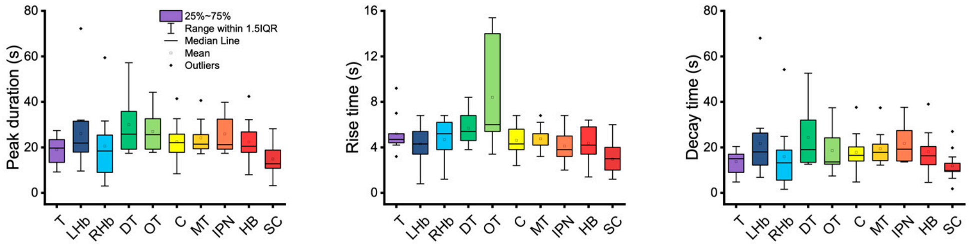

Figure S3. Box charts showing peak duration, rise and decay time for each of the ten brain regions (colored as in Figure 1a) during ictal regime. Peak duration and decay time are not significantly different between regions. Significant difference in rise time is limited to optic tectum (OT) with respect to spinal cord (SC). For p-values calculated with two-sample K-S test (Bonferroni correction α = 0.00111), see Table S5.

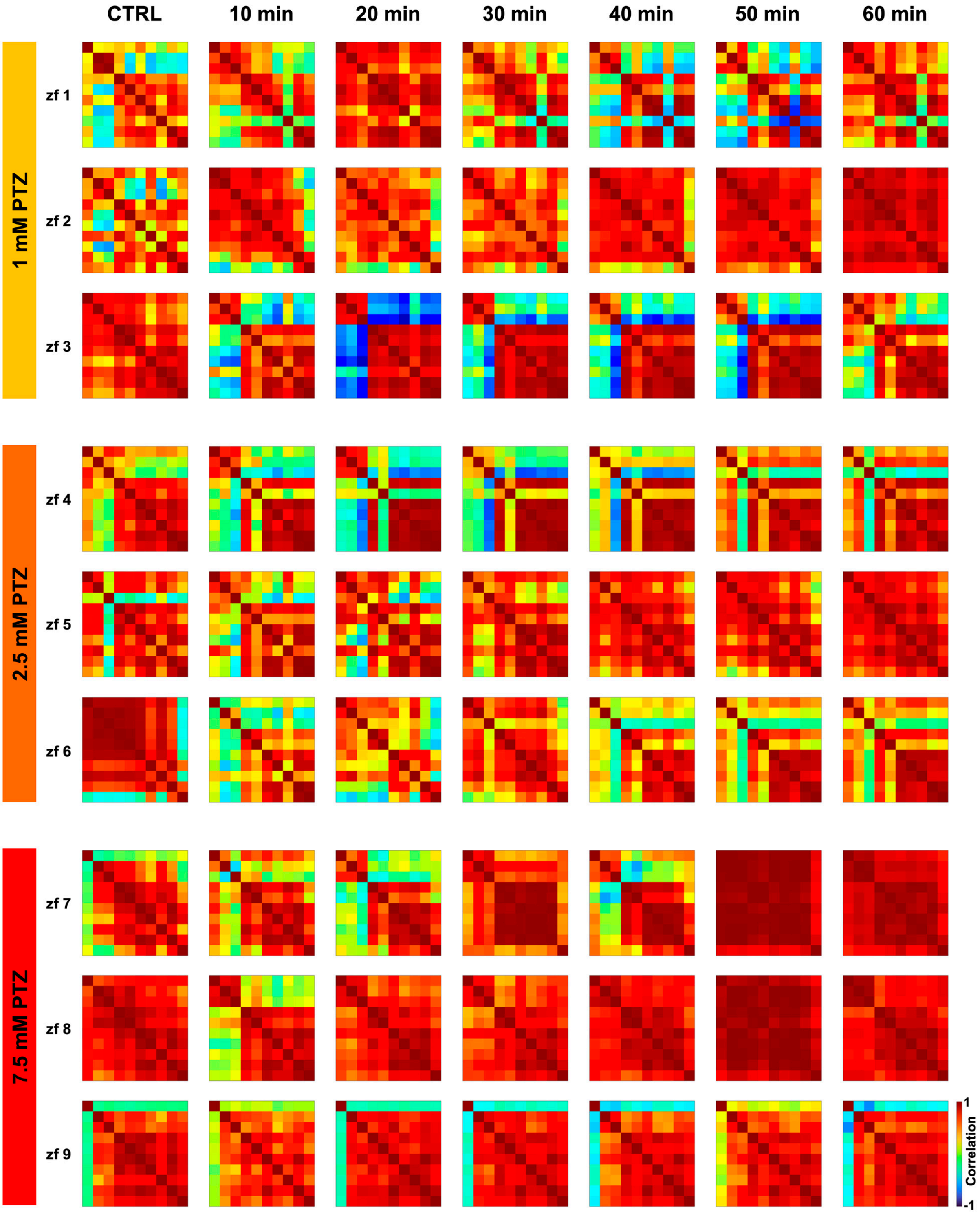

Figure S4. Correlation matrices reporting Pearson's correlation coefficients of neuronal activity over the time of exposure to submaximal PTZ concentrations (1.0, 2.5, and 7.5 mM) across brain regions as in Figure 2 (telencephalon, left habenula, right habenula, dorsal thalamus, optic tectum, cerebellum, medial tegmentum, interpeduncular nucleus, hindbrain, and spinal cord).

ACTIVITY PEAKS FREQUENCY - INTRAGROUP ANALYSIS - TWO-WAY ANOVA RM

Telencephalon

Comparison	p-value 1mM	2.5mM	7.5mM	15mM
ctrl 10min	0.61081	1	0.99999	0.94111
ctrl 20min	0.99996	0.99932	1	0.58012
ctrl 30min	0.90348	0.99829	0.99923	0.91294
ctrl 40min	0.99805	1	0.99587	0.41547
ctrl 50min	0.99666	0.99999	0.97457	0.97417
ctrl 60min	0.99956	0.69996	0.99996	1
10min 20min	0.75861	0.99992	1	0.98375
10min 30min	0.9959	0.99971	0.99998	1
10min 40min	0.87563	1	0.99961	0.92433
10min 50min	0.89578	0.99979	0.93176	1
10min 60min	0.82396	0.62293	1	0.94925
20min 30min	0.97	1	0.99989	0.99184
20min 40min	0.99997	0.99986	0.99889	0.9999
20min 50min	0.9999	0.99383	0.95143	0.95858
20min 60min	1	0.46031	1	0.59924
30min 40min	0.99456	0.99953	1	0.94999
30min 50min	0.99664	0.98887	0.84777	0.99996
30min 60min	0.98647	0.42062	1	0.92338
40min 50min	1	0.99988	0.77375	0.8653
40min 60min	1	0.6415	0.99985	0.43247
50min 60min	1	0.81075	0.9143	0.97875

Left Habenula

Comparison	p-value 1mM	2.5mM	7.5mM	15mM
ctrl 10min	0.97465	1	0.98232	0.42628
ctrl 20min	1	0.99864	0.95051	0.46672
ctrl 30min	0.91242	0.97841	0.18859	0.9432
ctrl 40min	0.99477	0.94962	0.11736	0.77269
ctrl 50min	0.86826	0.82511	0.9973	0.99501
ctrl 60min	0.98553	0.44729	0.19503	0.96459
10min 20min	0.98764	0.99887	0.99999	0.01985
10min 30min	0.99995	0.98035	0.52497	0.10051
10min 40min	0.99998	0.95313	0.3659	0.04957
10min 50min	0.9996	0.8323	0.99998	0.18473
10min 60min	1	0.45547	0.53758	0.896
20min 30min	0.94485	0.99984	0.63322	0.94841
20min 40min	0.99827	0.99845	0.46099	0.99722
20min 50min	0.91027	0.97397	0.99932	0.80446
20min 60min	0.99379	0.7173	0.6461	0.13298
30min 40min	0.99847	1	0.9999	0.99926
30min 50min	1	0.99806	0.40011	0.99958
30min 60min	0.99975	0.87785	1	0.50895
40min 50min	0.99497	0.99977	0.26664	0.97727
40min 60min	1	0.93211	0.99984	0.29868
50min 60min	0.99869	0.99049	0.41129	0.72994

Right Habenula

Comparison	p-value 1mM	2.5mM	7.5mM	15mM
ctrl 10min	0.99988	0.85622	0.75911	0.96144
ctrl 20min	0.90153	0.85923	0.99684	0.67187
ctrl 30min	0.46628	0.97245	0.70918	0.55512
ctrl 40min	0.9038	0.38478	0.43024	0.92226
ctrl 50min	0.85148	0.02103	1	0.34127
ctrl 60min	0.83674	0.00211	0.56802	0.37796
10min 20min	0.97657	1	0.96541	0.98995
10min 30min	0.64261	0.39899	1	0.96457
10min 40min	0.97745	0.05776	0.9958	1
10min 50min	0.95404	0.00269	0.83195	0.82987
10min 60min	0.94641	3.24E-04	0.99981	0.86465
20min 30min	0.97456	0.4025	0.94605	0.99999
20min 40min	1	0.05844	0.74293	0.99743
20min 50min	1	0.00272	0.99942	0.99469
20min 60min	1	3.27E-04	0.8646	0.99747
30min 40min	0.97359	0.84354	0.99833	0.9861
30min 50min	0.98877	0.08696	0.78753	0.99942
30min 60min	0.99122	0.00832	0.99997	0.99983
40min 50min	1	0.53153	0.50886	0.89513
40min 60min	1	0.07063	0.99997	0.92161
50min 60min	1	0.78254	0.6523	1

Dorsal Thalamus

Comparison	p-value 1mM	2.5mM	7.5mM	15mM
ctrl 10min	0.82395	0.91793	0.9979	0.99573
ctrl 20min	0.8756	0.91663	0.04105	0.00664
ctrl 30min	0.98629	0.07841	0.00352	0.01437
ctrl 40min	0.90991	0.04859	0.003	0.01851
ctrl 50min	0.3423	0.02345	0.00896	0.96034
ctrl 60min	0.82619	0.03897	0.00759	0.0021
10min 20min	1	1	0.09667	0.01772
10min 30min	0.99597	0.39442	0.00818	0.03874
10min 40min	0.99999	0.26849	0.00694	0.04988
10min 50min	0.96428	0.14036	0.02129	0.99984
10min 60min	1	0.22215	0.018	0.00541
20min 30min	0.99884	0.39642	0.74292	0.99884
20min 40min	1	0.27003	0.68735	0.99463
20min 50min	0.93759	0.14126	0.96249	0.03083
20min 60min	1	0.22348	0.93998	0.98835
30min 40min	0.99968	0.99992	1	1
30min 50min	0.74208	0.98768	0.99637	0.06707
30min 60min	0.99615	0.99934	0.99873	0.88476
40min 50min	0.90942	0.99922	0.99147	0.08595
40min 60min	0.99999	1	0.99636	0.81971
50min 60min	0.96339	0.9999	1	0.00931

Optic Tectum

Comparison	p-value 1mM	2.5mM	7.5mM	15mM
ctrl 10min	0.691	0.09851	0.50523	0.0019
ctrl 20min	0.44822	0.00984	0.00672	1.70E-04
ctrl 30min	0.1228	1.41E-04	0.00195	7.74E-05
ctrl 40min	0.02648	1.64E-05	0.00259	2.32E-04
ctrl 50min	0.03416	3.13E-05	0.0153	3.15E-04
ctrl 60min	0.01162	1.56E-05	0.0039	3.72E-06
10min 20min	0.99926	0.79495	0.15527	0.64887
10min 30min	0.81395	0.01495	0.0444	0.3346
10min 40min	0.3203	9.68E-04	0.05988	0.77411
10min 50min	0.3899	0.00221	0.31954	0.87507
10min 60min	0.15679	9.01E-04	0.09143	0.00519
20min 30min	0.96164	0.14676	0.98308	0.99575
20min 40min	0.53832	0.00869	0.99572	0.99998
20min 50min	0.62526	0.0213	0.99835	0.99911
20min 60min	0.29486	0.00803	0.99983	0.08083
30min 40min	0.95864	0.61498	1	0.9775
30min 50min	0.98233	0.88833	0.84968	0.93325
30min 60min	0.77592	0.58752	0.99919	0.20444
40min 50min	1	0.99745	0.91761	0.99999
40min 60min	0.99844	1	0.99996	0.05564
50min 60min	0.9933	0.99596	0.97573	0.03867

Cerebellum

Comparison	p-value 1mM	2.5mM	7.5mM	15mM
ctrl 10min	0.66001	0.99971	0.99987	0.99998
ctrl 20min	0.43363	0.99916	0.17997	0.0042
ctrl 30min	0.83133	0.61939	0.01436	0.01991
ctrl 40min	0.67694	0.1472	0.01974	0.26345
ctrl 50min	0.96844	0.19904	0.06049	0.92711
ctrl 60min	0.12153	0.12807	0.03331	0.76032
10min 20min	0.99948	1	0.286	0.00291
10min 30min	0.99986	0.8178	0.0245	0.01359
10min 40min	1	0.2541	0.0337	0.18882
10min 50min	0.98478	0.33357	0.10187	0.97593
10min 60min	0.83636	0.22355	0.05672	0.63279
20min 30min	0.98734	0.85195	0.71166	0.95584
20min 40min	0.99922	0.28232	0.81124	0.21793
20min 50min	0.89325	0.36773	0.99095	8.10E-04
20min 60min	0.96563	0.24912	0.93211	0.0467
30min 40min	0.99992	0.91213	0.99999	0.67517
30min 50min	0.99925	0.96305	0.9714	0.00349
30min 60min	0.66629	0.88026	0.99826	0.21081
40min 50min	0.98756	0.99999	0.99178	0.04997
40min 60min	0.82262	1	0.99988	0.95087
50min 60min	0.42534	0.99993	0.99974	0.2312

Table S1(I). Intra-group (same PTZ concentration) comparison of peak frequency at the different measured time points (Figure 1c), compared to pre-exposure (- 5 min) values. One-way repeated measures ANOVA followed by Tukey's test. Colored cells indicate p-values < 0.05.

ACTIVITY PEAKS FREQUENCY - INTRAGROUP ANALYSIS - TWO-WAY ANOVA RM

Medial Tegmentum

Comparison	p-value 1mM	2.5mM	7.5mM	15mM
ctrl 10min	0.92168	1	0.99987	0.58568
ctrl 20min	0.99842	1	0.35763	1.41E-04
ctrl 30min	0.99998	0.66064	0.00812	4.13E-04
ctrl 40min	0.99998	0.17463	0.01161	0.0057
ctrl 50min	0.99937	0.11386	0.00233	0.33975
ctrl 60min	0.98454	0.11581	0.01707	0.08215
10min 20min	0.99607	1	0.52109	0.00182
10min 30min	0.83078	0.66116	0.01373	0.00625
10min 40min	0.83228	0.17488	0.01971	0.10603
10min 50min	0.99257	0.11402	0.00385	0.99879
10min 60min	0.99987	0.11598	0.02903	0.78108
20min 30min	0.98737	0.69722	0.28141	0.98296
20min 40min	0.98766	0.19248	0.37512	0.24179
20min 50min	1	0.12612	0.08606	0.00383
20min 60min	0.99996	0.12827	0.49414	0.01806
30min 40min	1	0.92447	0.99999	0.61815
30min 50min	0.99275	0.81673	0.98274	0.0136
30min 60min	0.94454	0.82181	0.99909	0.06572
40min 50min	0.99294	0.99995	0.94506	0.21887
40min 60min	0.94534	0.99996	0.99998	0.67625
50min 60min	0.99981	1	0.86947	0.95606

IPN

Comparison	p-value 1mM	2.5mM	7.5mM	15mM
ctrl 10min	0.08612	0.99997	0.9945	0.01704
ctrl 20min	0.34503	0.99842	0.09757	1.29E-04
ctrl 30min	0.8221	0.64551	0.00865	1.07E-04
ctrl 40min	0.51505	0.97334	0.00745	0.00293
ctrl 50min	0.25323	0.80065	0.01865	0.05672
ctrl 60min	0.24831	0.57656	0.08291	0.00176
10min 20min	0.9596	0.99997	0.25253	0.07839
10min 30min	0.55461	0.78081	0.02434	0.06215
10min 40min	0.8538	0.99533	0.0209	0.92218
10min 50min	0.98971	0.90464	0.0527	0.98818
10min 60min	0.99072	0.7167	0.21841	0.78857
20min 30min	0.96609	0.89138	0.75774	1
20min 40min	0.99983	0.99976	0.70828	0.38764
20min 50min	0.99998	0.96797	0.94387	0.02367
20min 60min	0.99997	0.84239	1	0.56083
30min 40min	0.9969	0.97834	1	0.32286
30min 50min	0.90832	0.99993	0.99891	0.01868
30min 60min	0.90363	1	0.80888	0.48196
40min 50min	0.99684	0.99773	0.99706	0.56833
40min 60min	0.99639	0.95844	0.76284	0.99986
50min 60min	1	0.99948	0.96537	0.39405

Hindbrain

Comparison	p-value 1mM	2.5mM	7.5mM	15mM
ctrl 10min	0.94364	0.99999	1	0.7939
ctrl 20min	0.99756	0.99995	0.05845	0.00986
ctrl 30min	1	0.91689	0.0012	0.00149
ctrl 40min	1	0.81452	0.00245	0.00475
ctrl 50min	1	0.48481	0.00855	0.76619
ctrl 60min	0.9972	0.38771	0.01774	0.01716
10min 20min	0.99889	1	0.06552	0.09908
10min 30min	0.89011	0.83858	0.00133	0.01398
10min 40min	0.9452	0.70839	0.00274	0.04765
10min 50min	0.92073	0.38054	0.00958	1
10min 60min	0.99906	0.29717	0.01992	0.16718
20min 30min	0.98923	0.80831	0.27616	0.88681
20min 40min	0.99772	0.67203	0.49476	0.99911
20min 50min	0.99472	0.35037	0.89657	0.10806
20min 60min	1	0.27181	0.98869	0.99983
30min 40min	1	0.99996	0.99891	0.987
30min 50min	1	0.9728	0.86444	0.0153
30min 60min	0.98812	0.93258	0.63745	0.72937
40min 50min	1	0.99554	0.98301	0.05213
40min 60min	0.99737	0.98168	0.87381	0.98245
50min 60min	0.99406	0.99999	0.99918	0.1815

Spinal cord

Comparison	p-value 1mM	2.5mM	7.5mM	15mM
ctrl 10min	0.86369	0.99948	0.98584	0.96151
ctrl 20min	0.98636	0.99701	0.9974	9.37E-04
ctrl 30min	0.99889	0.99963	0.33425	3.18E-04
ctrl 40min	1	0.95344	0.7214	0.0075
ctrl 50min	0.99768	0.85866	0.66079	0.62835
ctrl 60min	1	0.92153	0.64793	0.61721
10min 20min	0.99836	1	0.84264	0.0039
10min 30min	0.62236	0.98153	0.11086	0.00122
10min 40min	0.91519	0.80771	0.32354	0.03457
10min 50min	0.5844	0.64912	0.2794	0.983
10min 60min	0.86968	0.74524	0.27079	0.98079
20min 30min	0.87728	0.95764	0.61882	0.98633
20min 40min	0.9954	0.72991	0.94657	0.82768
20min 50min	0.84987	0.56312	0.91654	0.01387
20min 60min	0.98766	0.66177	0.90923	0.01433
30min 40min	0.9953	0.99638	0.98804	0.42383
30min 50min	1	0.97027	0.99485	0.00411
30min 60min	0.99868	0.99004	0.99578	0.00424
40min 50min	0.99187	0.99992	1	0.12301
40min 60min	1	1	1	0.12682
50min 60min	0.99731	1	1	1

Table S1(II). Intra-group (same PTZ concentration) comparison of peak frequency at the different measured time points (Figure 1c), compared to pre-exposure (- 5 min) values. One-way repeated measures ANOVA followed by Tukey’s test. Colored cells indicate p-values < 0.05.

ACTIVITY PEAKS FREQUENCY - INTERGROUP ANALYSIS - ONE-WAY ANOVA RM						
		Telencephalon	Left Habenula	Right Habenula	Dorsal Thalamus	Optic Tectum
Time point	Comparison	p-value	p-value	p-value	p-value	p-value
ctrl	1mM 2.5mM	0.96537	0.95591	1	0.94593	0.89443
	1mM 7.5mM	0.9218	1	0.99993	0.99902	0.93239
	1mM 15mM	0.27078	0.99986	0.99732	0.32425	0.44835
	2.5mM 7.5mM	0.9985	0.95491	0.99996	0.97624	0.99954
	2.5mM 15mM	0.52805	0.93647	0.99698	0.64821	0.8588
	7.5mM 15mM	0.62813	0.99989	0.99426	0.39844	0.806
10min	1mM 2.5mM	0.9677	0.76155	0.94789	0.9801	0.35235
	1mM 7.5mM	0.99947	0.99983	0.51321	0.99072	0.08155
	1mM 15mM	0.97989	0.98844	0.79735	0.71695	0.00655
	2.5mM 7.5mM	0.98576	0.80394	0.229	0.90253	0.86226
	2.5mM 15mM	0.83199	0.56707	0.47063	0.90781	0.3011
	7.5mM 15mM	0.95825	0.97827	0.96461	0.53347	0.75803
20min	1mM 2.5mM	0.99872	0.99632	0.80851	0.96368	0.11632
	1mM 7.5mM	0.9392	0.97242	0.99911	0.02176	4.07E-07
	1mM 15mM	0.90168	0.89902	0.44142	0.00908	0.00104
	2.5mM 7.5mM	0.97443	0.91472	0.73393	0.06984	9.86E-04
	2.5mM 15mM	0.95028	0.96398	0.09073	0.03224	0.30152
	7.5mM 15mM	0.99949	0.6748	0.52289	0.98815	0.11249
30min	1mM 2.5mM	0.98855	0.94946	0.94594	0.09019	0.00529
	1mM 7.5mM	0.98779	0.88717	0.86768	6.82E-05	3.52E-08
	1mM 15mM	0.92191	0.79727	0.49049	0.00692	0.00237
	2.5mM 7.5mM	0.91622	0.59177	0.55476	0.07521	0.00696
	2.5mM 15mM	0.77828	0.98152	0.21156	0.74279	0.99291
	7.5mM 15mM	0.99021	0.3675	0.9133	0.46206	0.01476
40min	1mM 2.5mM	0.99835	0.99908	0.99519	0.10223	0.00116
	1mM 7.5mM	0.88242	0.65436	0.37094	1.20E-04	1.21E-06
	1mM 15mM	0.97987	0.86014	0.86366	0.01835	0.04142
	2.5mM 7.5mM	0.94073	0.5693	0.50945	0.09625	0.18882
	2.5mM 15mM	0.99606	0.91502	0.94858	0.89039	0.58275
	7.5mM 15mM	0.98549	0.22253	0.82881	0.35745	0.00923
50min	1mM 2.5mM	0.99267	0.99603	0.85377	0.26411	0.00248
	1mM 7.5mM	0.99806	0.97494	0.9869	0.01289	2.38E-04
	1mM 15mM	0.71171	0.83275	0.11497	0.94135	0.04351
	2.5mM 7.5mM	0.96936	0.99729	0.66968	0.54021	0.87814
	2.5mM 15mM	0.85807	0.92458	0.45252	0.57943	0.7234
	7.5mM 15mM	0.60429	0.9738	0.05468	0.05455	0.28989
60min	1mM 2.5mM	0.77892	0.94194	0.59444	0.11471	0.00277
	1mM 7.5mM	0.93387	0.79619	0.5627	0.00135	1.43E-05
	1mM 15mM	0.38004	0.99998	0.14719	0.00388	5.23E-04
	2.5mM 7.5mM	0.98435	0.98493	0.99995	0.34788	0.37836
	2.5mM 15mM	0.90915	0.93104	0.79943	0.55176	0.94935
	7.5mM 15mM	0.73716	0.77639	0.82553	0.98512	0.70313

ACTIVITY PEAKS FREQUENCY - INTERGROUP ANALYSIS						
		Cerebellum	Medial Tegmentum	IPN	Hindbrain	Spinal cord
Time point	Comparison	p-value	p-value	p-value	p-value	p-value
ctrl	1mM 2.5mM	0.88179	0.99439	0.94985	0.95058	0.97294
	1mM 7.5mM	0.94296	0.97806	0.99537	0.99993	0.68243
	1mM 15mM	0.13894	0.99737	0.98188	0.99825	0.95059
	2.5mM 7.5mM	0.99806	0.9147	0.99042	0.96435	0.90269
	2.5mM 15mM	0.46961	0.97028	0.79942	0.89562	0.99961
	7.5mM 15mM	0.36934	0.99686	0.92856	0.99576	0.93716
10min	1mM 2.5mM	0.98013	0.97319	0.78332	0.48641	0.9381
	1mM 7.5mM	0.99339	0.83968	0.86299	0.89741	0.98819
	1mM 15mM	0.4836	0.99556	0.97258	0.99594	0.98845
	2.5mM 7.5mM	0.99935	0.97786	0.99852	0.88311	0.99409
	2.5mM 15mM	0.72141	0.91118	0.52217	0.35778	0.80536
	7.5mM 15mM	0.64782	0.71382	0.62185	0.79289	0.91719
20min	1mM 2.5mM	0.99158	1	0.99237	0.64879	0.97698
	1mM 7.5mM	0.06086	0.7385	0.13395	0.06726	0.61567
	1mM 15mM	5.66E-04	0.00103	0.03313	0.13358	0.00942
	2.5mM 7.5mM	0.11472	0.73362	0.07389	0.00299	0.37205
	2.5mM 15mM	0.0014	0.00101	0.01613	0.00754	0.00291
	7.5mM 15mM	0.35361	0.01981	0.9265	0.98868	0.17336
30min	1mM 2.5mM	0.42463	0.31615	0.7393	0.91248	0.76111
	1mM 7.5mM	2.70E-04	0.00821	7.00E-04	3.08E-05	0.00424
	1mM 15mM	0.00127	0.00111	0.0076	0.00827	3.51E-04
	2.5mM 7.5mM	0.02537	0.37879	0.01419	2.76E-04	0.0572
	2.5mM 15mM	0.08143	0.11476	0.09818	0.04614	0.00702
	7.5mM 15mM	0.96171	0.90704	0.8573	0.30003	0.85138
40min	1mM 2.5mM	0.11985	0.05655	0.99881	0.86268	0.71133
	1mM 7.5mM	7.35E-04	0.01324	0.00131	1.82E-04	0.06744
	1mM 15mM	0.02409	0.02423	0.31759	0.0333	0.02519
	2.5mM 7.5mM	0.24554	0.93975	0.00206	0.00216	0.46535
	2.5mM 15mM	0.90148	0.98578	0.39624	0.18539	0.25361
	7.5mM 15mM	0.62762	0.99575	0.12683	0.30307	0.97722
50min	1mM 2.5mM	0.09128	0.0864	0.99567	0.48858	0.3517
	1mM 7.5mM	0.00269	0.00466	0.01582	0.0013	0.02037
	1mM 15mM	0.59559	0.78221	0.9824	0.74337	0.31246
	2.5mM 7.5mM	0.54147	0.67259	0.02879	0.06505	0.53474
	2.5mM 15mM	0.66558	0.45741	0.99899	0.97546	0.99983
	7.5mM 15mM	0.07535	0.05638	0.04088	0.02354	0.58266
60min	1mM 2.5mM	0.27467	0.13817	0.9562	0.64852	0.61194
	1mM 7.5mM	0.00903	0.09614	0.14029	0.01444	0.0422
	1mM 15mM	0.24461	0.55518	0.37371	0.19317	0.47837
	2.5mM 7.5mM	0.44754	0.99809	0.34489	0.20982	0.44355
	2.5mM 15mM	0.99988	0.81562	0.68149	0.82764	0.99639
	7.5mM 15mM	0.48855	0.71908	0.94205	0.6763	0.57539

Table S2. Inter-group (different PTZ concentrations) comparison of peak frequency at the same measured time points (Figure 1c). Two-way repeated measures ANOVA followed by Tukey's test. Colored cells indicate p-values < 0.05.

REGIME PEAK FREQUENCY - INTER-REGIME ANALYSIS - ONE-WAY ANOVA RM	
Comparison	p-value
ctrl pre	0.02044
ctrl ictal	0.00403
ctrl post	0.12541
pre ictal	2.12E-04
pre post	0.0018
ictal post	0.06197

Table S3. Inter-regime comparison of peak frequency in the different brain activity regimes at 15 mM exposure (Figure 2b). One-way repeated measures ANOVA followed by Tukey's test. Colored cells indicate p-values < 0.05.

ACTIVITY PEAKS ICTAL - INTER-REGIME ANALYSIS - TWO-SAMPLE KOLMOGOROV-SMIRNOV TEST			
Bonferroni correction	alfa = 0.05/6 = 0.00833		

Peak duration	D	Z	Asymp prob > |D|
CTRL - PRE	0.20657	4.87084	4.05E-21
CTRL - ICTAL	0.82819	7.82814	6.61E-54
CTRL - POST	0.39016	7.85789	2.78E-54
PRE - ICTAL	0.87312	8.63938	2.08E-65
PRE - POST	0.23828	6.18107	1.02E-33
ICTAL - POST	0.88878	8.52065	9.93E-64

Peak prominence	D	Z	Asymp prob > |D|
CTRL - PRE	0.06735	1.58802	1.21E-02
CTRL - ICTAL	0.87459	8.26677	4.72E-60
CTRL - POST	0.08029	1.61705	0.00963
PRE - ICTAL	0.8842	8.7491	4.56E-67
PRE - POST	0.11458	2.97231	3.76E-08
ICTAL - POST	0.87132	8.35331	2.85E-61

Rise time	D	Z	Asymp prob > |D|
CTRL - PRE	0.11362	2.67922	1.04E-06
CTRL - ICTAL	0.59248	5.60024	7.55E-28
CTRL - POST	0.28717	5.78362	1.21E-29
PRE - ICTAL	0.63175	6.25108	1.78E-34
PRE - POST	0.23322	6.04991	2.53E-32
ICTAL - POST	0.69997	6.71057	9.90E-40

Decay time	D	Z	Asymp prob > |D|
CTRL - PRE	0.22937	5.40844	6.29E-26
CTRL - ICTAL	0.82949	7.84049	4.49E-54
CTRL - POST	0.32874	6.62081	1.09E-38
PRE - ICTAL	0.86992	8.60778	6.20E-65
PRE - POST	0.18273	4.74018	5.02E-20
ICTAL - POST	0.88616	8.49556	2.34E-63

Table S4. Inter-regime comparison of activity peak features (duration, rise/decay time and prominence) distributions (Figure 2d and S2). Two-samples K-S test with Bonferroni correction. Colored cells indicate p-values < 0.00833.

ACTIVITY PEAKS ICTAL - INTER-REGION ANALYSIS - TWO-SAMPLE KOLMOGOROV-SMIRNOV TEST			
Bonferroni correction	alfa = 0.05/45 = 0.00111		

Peak duration	D	Z	Asymp prob > |D|
T - LHb	0.33333	0.7785	0.47696
T - RHb	0.27273	0.62419	0.69158
T -DT	0.44444	0.9673	0.25153
T - OT	0.44444	0.9673	0.25153
T - C	0.3	0.67082	0.78693
T - MT	0.36667	0.79802	0.44246
T - IPN	0.38889	0.84639	0.34287
T - HB	0.2	0.44721	0.99446
T - SC	0.2	0.44721	0.99446
LHb - RHb	0.29545	0.70781	0.59575
LHb -DT	0.36111	0.81892	0.42716
LHb - OT	0.25	0.56695	0.85058
LHb - C	0.13333	0.3114	0.9996
LHb - MT	0.19444	0.44096	0.96849
LHb - IPN	0.30556	0.69293	0.63835
LHb - HB	0.15	0.35032	0.99644
LHb - SC	0.52564	1.31305	0.03971
RHb -DT	0.45455	1.0113	0.18939
RHb - OT	0.45455	1.0113	0.18939
RHb - C	0.26364	0.60338	0.73705
RHb - MT	0.36364	0.80904	0.41272
RHb - IPN	0.45455	1.0113	0.18939
RHb - HB	0.26364	0.60338	0.73705
RHb - SC	0.32867	0.80228	0.42689
DT - OT	0.22222	0.4714	0.98947
DT - C	0.25556	0.5562	0.83428
DT - MT	0.33333	0.70711	0.73011
DT - IPN	0.22222	0.4714	0.98947
DT - HB	0.34444	0.74966	0.53859
DT - SC	0.69231	1.59654	0.00653
OT - C	0.25556	0.5562	0.83428
OT - MT	0.22222	0.4714	0.98947
OT - IPN	0.22222	0.4714	0.98947
OT - HB	0.27778	0.60456	0.73958
OT - SC	0.69231	1.59654	0.00653
C - MT	0.2	0.43529	0.95726
C - IPN	0.28889	0.62875	0.69136
C - HB	0.1	0.22361	1
C - SC	0.49231	1.17043	0.09426
MT - IPN	0.22222	0.4714	0.98947
MT - HB	0.27778	0.60456	0.73958
MT - SC	0.69231	1.59654	0.00653
IPN - HB	0.38889	0.84639	0.34287
IPN - SC	0.69231	1.59654	0.00653
HB - SC	0.49231	1.17043	0.09426

Peak prominence	D	Z	Asymp prob > |D|
T - LHb	0.63333	1.47915	0.01531
T - RHb	0.43636	0.9987	0.19553
T -DT	0.9	1.95879	2.17E-04
T - OT	0.9	1.95879	2.17E-04
T - C	0.9	2.01246	2.17E-04
T - MT	0.9	1.95879	2.17E-04
T - IPN	0.9	1.95879	2.17E-04
T - HB	0.9	2.01246	2.17E-04
T - SC	0.41538	0.98755	0.22064
LHb - RHb	0.27273	0.65336	0.69387
LHb -DT	0.72222	1.63785	0.00462
LHb - OT	0.66667	1.51186	0.01197
LHb - C	0.66667	1.557	0.00673
LHb - MT	0.83333	1.88982	4.42E-04
LHb - IPN	0.83333	1.88982	4.42E-04
LHb - HB	0.66667	1.557	0.00673
LHb - SC	0.41667	1.04083	0.18481
RHb -DT	0.79798	1.77539	0.00162
RHb - OT	0.77778	1.73045	0.00226
RHb - C	0.63636	1.45644	0.01115
RHb - MT	0.81818	1.82034	7.62E-04
RHb - IPN	0.81818	1.82034	7.62E-04
RHb - HB	0.63636	1.45644	0.01115
RHb - SC	0.45455	1.10953	0.11845
DT - OT	0.22222	0.4714	0.98947
DT - C	0.58889	1.28167	0.04456
DT - MT	0.22222	0.4714	0.98947
DT - IPN	0.22222	0.4714	0.98947
DT - HB	0.68889	1.49932	0.01234
DT - SC	1	2.30612	4.02E-06
OT - C	0.67778	1.47514	0.01764
OT - MT	0.22222	0.4714	0.98947
OT - IPN	0.22222	0.4714	0.98947
OT - HB	0.77778	1.69278	0.0037
OT - SC	1	2.30612	4.02E-06
C - MT	0.58889	1.28167	0.04456
C - IPN	0.58889	1.28167	0.04456
C - HB	0.4	0.89443	0.41752
C - SC	1	2.37743	1.75E-06
MT - IPN	0.22222	0.4714	0.98947
MT - HB	0.67778	1.47514	0.01764
MT - SC	1	2.30612	4.02E-06
IPN - HB	0.67778	1.47514	0.01764
IPN - SC	1	2.30612	4.02E-06
HB - SC	1	2.37743	1.75E-06

Table S5(I). Inter-region comparison of activity peak features (duration and rise/decay time) during ictal regime (Figures 2e and S3). Two-samples K-S test with Bonferroni correction. Colored cells indicate p-values < 0.00111.

ACTIVITY PEAKS ICTAL - INTER-REGION ANALYSIS - TWO-SAMPLE KOLMOGOROV-SMIRNOV TEST							
Bonferroni correction alfa = 0.05/45 = 0.00111							
Rise time	D	Z	Asymp prob > |D|	Decay time	D	Z	Asymp prob > |D|
T - LHb	0.31667	0.73957	0.55772	T - LHb	0.38333	0.89527	0.31827
T - RHb	0.2	0.45774	0.94979	T - RHb	0.18182	0.41613	0.9707
T -DT	0.35556	0.77384	0.49217	T -DT	0.44444	0.9673	0.25153
T - OT	0.57778	1.25749	0.06034	T - OT	0.33333	0.72548	0.57507
T - C	0.4	0.89443	0.41752	T - C	0.3	0.67082	0.78693
T - MT	0.35556	0.77384	0.49217	T - MT	0.35556	0.77384	0.49217
T - IPN	0.45556	0.99148	0.23007	T - IPN	0.44444	0.9673	0.25153
T - HB	0.4	0.89443	0.41752	T - HB	0.3	0.67082	0.78693
T - SC	0.66923	1.59105	0.00663	T - SC	0.36923	0.87782	0.32743
LHb - RHb	0.38636	0.92559	0.28628	LHb - RHb	0.31061	0.7441	0.51621
LHb -DT	0.38889	0.88192	0.34458	LHb -DT	0.25	0.56695	0.85058
LHb - OT	0.52778	1.19689	0.07849	LHb - OT	0.30556	0.69293	0.63835
LHb - C	0.16667	0.38925	0.98909	LHb - C	0.2	0.4671	0.95279
LHb - MT	0.19444	0.44096	0.96849	LHb - MT	0.25	0.56695	0.85058
LHb - IPN	0.19444	0.44096	0.96849	LHb - IPN	0.25	0.56695	0.85058
LHb - HB	0.16667	0.38925	0.98909	LHb - HB	0.16667	0.38925	0.98909
LHb - SC	0.44872	1.1209	0.10834	LHb - SC	0.51923	1.29704	0.04883
RHb -DT	0.24242	0.53936	0.86122	RHb -DT	0.39394	0.87646	0.32443
RHb - OT	0.41414	0.92141	0.28157	RHb - OT	0.27273	0.60678	0.73808
RHb - C	0.24545	0.56177	0.81399	RHb - C	0.34545	0.79064	0.43451
RHb - MT	0.28283	0.62925	0.68808	RHb - MT	0.36364	0.80904	0.41272
RHb - IPN	0.32323	0.71915	0.58659	RHb - IPN	0.54545	1.21356	0.06961
RHb - HB	0.31818	0.72822	0.56769	RHb - HB	0.26364	0.60338	0.73705
RHb - SC	0.57343	1.39972	0.0259	RHb - SC	0.31469	0.76814	0.46488
DT - OT	0.33333	0.70711	0.73011	DT - OT	0.22222	0.4714	0.98947
DT - C	0.38889	0.84639	0.34287	DT - C	0.25556	0.5562	0.83428
DT - MT	0.44444	0.94281	0.35171	DT - MT	0.22222	0.4714	0.98947
DT - IPN	0.44444	0.94281	0.35171	DT - IPN	0.33333	0.70711	0.73011
DT - HB	0.5	1.08821	0.10558	DT - HB	0.3	0.65293	0.63748
DT - SC	0.73504	1.6951	0.00296	DT - SC	0.5812	1.34031	0.03743
OT - C	0.47778	1.03985	0.16782	OT - C	0.35556	0.77384	0.49217
OT - MT	0.55556	1.17851	0.12587	OT - MT	0.33333	0.70711	0.73011
OT - IPN	0.66667	1.41421	0.03357	OT - IPN	0.44444	0.94281	0.35171
OT - HB	0.45556	0.99148	0.23007	OT - HB	0.25556	0.5562	0.83428
OT - SC	0.81197	1.87249	6.19E-04	OT - SC	0.4359	1.00523	0.1987
C - MT	0.27778	0.60456	0.73958	C - MT	0.2	0.43529	0.95726
C - IPN	0.23333	0.50783	0.90384	C - IPN	0.25556	0.5562	0.83428
C - HB	0.3	0.67082	0.78693	C - HB	0.1	0.22361	1
C - SC	0.59231	1.40817	0.02434	C - SC	0.56923	1.35331	0.0328
MT - IPN	0.33333	0.70711	0.73011	MT - IPN	0.22222	0.4714	0.98947
MT - HB	0.27778	0.60456	0.73958	MT - HB	0.2	0.43529	0.95726
MT - SC	0.61538	1.41915	0.02196	MT - SC	0.5812	1.34031	0.03743
IPN - HB	0.28889	0.62875	0.69136	IPN - HB	0.3	0.65293	0.63748
IPN - SC	0.39316	0.90668	0.29614	IPN - SC	0.76923	1.77394	0.00155
HB - SC	0.59231	1.40817	0.02434	HB - SC	0.46923	1.11556	0.11445

Table S5(II). Inter-region comparison of activity peak features (duration and rise/decay time) during ictal regime (Figures 2e and S3). Two-samples K-S test with Bonferroni correction. Colored cells indicate p-values < 0.00111.

BEHAVIORAL PARAMETERS - INTRAGROUP ANALYSIS - ONE-WAY ANOVA RM				
Comparison	p-value			
Distance traveled	1 mM	2.5 mM	7.5 mM	15 mM
ctrl - 30min	0.00137	0.04021	0.03152	7.08E-04
ctrl - 1h	9.99E-06	2.01E-04	6.76E-04	0.35496
30min - 1h	2.32E-04	0.00203	0.01454	0.00233
Max speed	1 mM	2.5 mM	7.5 mM	15 mM
ctrl - 30min	0.98267	0.00323	0.00937	0.00717
ctrl - 1h	7.87957	0.00551	0.00715	0.00868
30min - 1h	6.8969	0.84177	0.96268	0.98082
Max acceleration	1 mM	2.5 mM	7.5 mM	15 mM
ctrl - 30min	0.61354	0.00135	0.02347	0.00191
ctrl - 1h	0.00461	9.02E-04	0.01162	0.00103
30min - 1h	0.01245	0.86324	0.80832	0.73692
Bout duration	1 mM	2.5 mM	7.5 mM	15 mM
ctrl - 30min	0.87585	4.22E-04	1.01E-05	9.36E-05
ctrl - 1h	0.70748	2.06E-04	1.23E-05	2.12E-05
30min - 1h	0.9455	0.53473	0.85965	0.04556
Bout frequency	1 mM	2.5 mM	7.5 mM	15 mM
ctrl - 30min	0.10342	6.96E-05	7.96E-06	2.60E-04
ctrl - 1h	0.06409	7.67E-05	1.00E-05	2.30E-04
30min - 1h	0.92502	0.98009	0.8077	0.97785
Bout distance	1 mM	2.5 mM	7.5 mM	15 mM
ctrl - 30min	0.90298	0.00337	0.00406	2.32E-04
ctrl - 1h	0.03478	0.0012	0.01645	3.98E-04
30min - 1h	0.05911	0.48606	0.41149	0.69106
% movement	1 mM	2.5 mM	7.5 mM	15 mM
ctrl - 30min	0.32718	0.30744	0.17733	0.01569
ctrl - 1h	0.28261	0.81089	0.3762	0.0542
30min - 1h	0.99184	0.6063	0.81712	0.57287

Table S6. Intra-group (same PTZ concentration) comparison of swimming kinematic features (distance traveled, percentage of time spent in movement, bout max velocity, bout max acceleration, bout, bout duration, bout displacement and bout frequency) presented in Figures 3c, d, f-j, compared to pre-exposure (CTRL) values. One-way repeated measures ANOVA followed by Tukey's test. Colored cells indicate p-values < 0.05.

BEHAVIORAL PARAMETERS - INTERGROUP ANALYSIS - TWO-WAY ANOVA RM			
Comparison	p-value		
Distance traveled	CTRL	30min	1h
1 mM - 2.5 mM	0.99806	0.70377	5.94E-04
1 mM - 7.5 mM	0.62978	0.00104	3.33E-07
1 mM - 15 mM	0.78609	0.00296	0.02797
2.5 mM - 7.5 mM	0.7335	0.01444	0.01862
2.5 mM - 15 mM	0.87089	0.03753	4.15E-07
7.5 mM - 15 mM	0.993	0.9742	6.98E-08
Max speed	CTRL	30min	1h
1 mM - 2.5 mM	0.99429	1.43E-04	0.01081
1 mM - 7.5 mM	0.99761	1.35E-06	8.35E-06
1 mM - 15 mM	0.79683	2.04E-05	6.47E-04
2.5 mM - 7.5 mM	0.97121	0.24238	0.03625
2.5 mM - 15 mM	0.65299	0.85892	0.66382
7.5 mM - 15 mM	0.88479	0.66798	0.31712
Max acceleration	CTRL	30min	1h
1 mM - 2.5 mM	0.98709	0.00657	0.18286
1 mM - 7.5 mM	0.99994	1.29E-05	8.76E-05
1 mM - 15 mM	0.92722	0.00828	0.1929
2.5 mM - 7.5 mM	0.9799	0.08258	0.01529
2.5 mM - 15 mM	0.78173	0.99967	0.99999
7.5 mM - 15 mM	0.94365	0.06778	0.01424
Bout duration	CTRL	30min	1h
1 mM - 2.5 mM	0.99352	6.94E-08	0
1 mM - 7.5 mM	0.99935	1.94E-07	2.86E-07
1 mM - 15 mM	0.99754	1.60E-06	8.14E-08
2.5 mM - 7.5 mM	0.99896	0.11513	0.00224
2.5 mM - 15 mM	0.99985	0.01026	0.0435
7.5 mM - 15 mM	0.99988	0.68646	0.59424

Comparison	p-value		
Bout frequency	CTRL	30min	1h
1 mM - 2.5 mM	0.48456	0	0
1 mM - 7.5 mM	0.93265	0	0
1 mM - 15 mM	0.93067	0	0
2.5 mM - 7.5 mM	0.20156	1	0.99658
2.5 mM - 15 mM	0.19936	0.95853	0.9999
7.5 mM - 15 mM	1	0.96286	0.99883
Bout distance	CTRL	30min	1h
1 mM - 2.5 mM	1	2.74E-05	2.09E-06
1 mM - 7.5 mM	0.99976	4.34E-07	2.33E-06
1 mM - 15 mM	0.99538	0.0013	0.00553
2.5 mM - 7.5 mM	0.99981	0.00666	0.99996
2.5 mM - 15 mM	0.99572	0.42446	0.01832
7.5 mM - 15 mM	0.99882	1.42E-04	0.02034
% movement	CTRL	30min	1h
1 mM - 2.5 mM	0.99696	0.02544	0.2519
1 mM - 7.5 mM	0.90673	0.00229	0.01218
1 mM - 15 mM	0.51198	0.01593	0.02491
2.5 mM - 7.5 mM	0.9652	0.74488	0.4648
2.5 mM - 15 mM	0.63507	0.99688	0.65155
7.5 mM - 15 mM	0.88513	0.85105	0.98935

Table S7. Inter-group (different PTZ concentrations) comparison of swimming kinematic features (distance traveled, percentage of time spent in movement, bout max velocity, bout max acceleration, bout, bout duration, bout displacement and bout frequency) presented in Figures 3c, d, f-j, at 60-minute PTZ exposure. Two-way repeated measures ANOVA followed by Tukey's test. Colored cells indicate p-values < 0.05.

Video S1. Exploratory behavior in physiological conditions (CTRL).

Video S2. Swimming behavior after 60-minute exposure to 1 mM PTZ.

Video S3. Eye movement behavior after 30-minute exposure to 2.5 mM PTZ.

Video S4. Swimming behavior after 60-minute exposure to 7.5 mM PTZ.

Video S5. Swimming behavior after 60-minute exposure to 15 mM PTZ.
